# PINK1 and Parkin regulate IP_3_R-mediated ER calcium release

**DOI:** 10.1038/s41467-023-40929-z

**Published:** 2023-08-25

**Authors:** Su Jin Ham, Heesuk Yoo, Daihn Woo, Da Hyun Lee, Kyu-Sang Park, Jongkyeong Chung

**Affiliations:** 1 Institute of Molecular Biology and Genetics, Seoul National University, Seoul, 08826 Republic of Korea; 2 Interdisciplinary Graduate Program in Genetic Engineering, Seoul National University, Seoul, 08826 Republic of Korea; 3 School of Biological Sciences, Seoul National University, Seoul, 08826 Republic of Korea; 4 Department of Physiology, Yonsei University Wonju College of Medicine, Wonju, 26426 Republic of Korea; 5 Mitohormesis Research Center, Yonsei University Wonju College of Medicine, Wonju, 26426 Republic of Korea

**Keywords:** Mitophagy, Calcium signalling, Endoplasmic reticulum

## Abstract

Although defects in intracellular calcium homeostasis are known to play a role in the pathogenesis of Parkinson’s disease (PD), the underlying molecular mechanisms remain unclear. Here, we show that loss of PTEN-induced kinase 1 (PINK1) and Parkin leads to dysregulation of inositol 1,4,5-trisphosphate receptor (IP_3_R) activity, robustly increasing ER calcium release. In addition, we identify that CDGSH iron sulfur domain 1 (CISD1, also known as mitoNEET) functions downstream of Parkin to directly control IP_3_R. Both genetic and pharmacologic suppression of CISD1 and its *Drosophila* homolog CISD (also known as Dosmit) restore the increased ER calcium release in PINK1 and Parkin null mammalian cells and flies, respectively, demonstrating the evolutionarily conserved regulatory mechanism of intracellular calcium homeostasis by the PINK1-Parkin pathway. More importantly, suppression of CISD in PINK1 and Parkin null flies rescues PD-related phenotypes including defective locomotor activity and dopaminergic neuronal degeneration. Based on these data, we propose that the regulation of ER calcium release by PINK1 and Parkin through CISD1 and IP_3_R is a feasible target for treating PD pathogenesis.

## Abstract (web summary)

Loss of two PD genes, PINK1 and Parkin, leads to a robust increase in ER calcium release. Here, the authors show that suppression of IP_3_R activity via inhibiting CISD1 is sufficient to rescue the PD-related phenotypes in PINK1 or Parkin null animal models.

## Introduction

Selective degeneration of dopaminergic (DA) neurons in the substantia nigra is responsible for the motor symptoms of Parkinson’s disease (PD). DA neurons are autonomous pacemakers that generate action potentials, accompanied by slow oscillations in intracellular calcium concentrations caused by calcium uptake through plasma membrane calcium channels and calcium release from the endoplasmic reticulum (ER)^1^. Losing control of these oscillations leaves neurons more vulnerable to stresses linked to PD such as ER stress, oxidative stress, inflammation, mitochondrial dysfunction, dysregulated autophagy, and defective calcium signaling. Interestingly, previous studies have reported that DA neurons under aging or PD conditions exhibit an increased reliance on plasma membrane and ER calcium channels as well as a sustained increase in cytosolic calcium levels^2,3^. The need to pump out excess calcium causes these DA neurons to demand higher energy inputs, leading to elevated mitochondrial calcium levels and oxidative phosphorylation (OXPHOS) rates which may result in irreversible mitochondrial damages that ultimately lead to cell death.

Furthermore, several PD-associated proteins including PTEN-induced kinase 1 (PINK1), Parkin, leucine-rich repeat kinase 2 (LRRK2), and alpha-synuclein have been shown to be involved in controlling cellular calcium homeostasis^4–9^. Previous studies have reported that PINK1 directly regulates leucine zipper-EF-hand-containing transmembrane protein 1 (LETM1)^4^, a mitochondrial inner membrane protein proposed as a proton-dependent calcium exchanger, and also controls calcium efflux from a mitochondrial Na^+^/Ca^2+^ exchanger^5^. Consequently, PINK1 deficiency leads to increased mitochondrial calcium levels and sensitivity to calcium-induced mitochondrial permeability transition, elevating cell death susceptibility^4,5^. In addition, the PINK1-Parkin pathway has been implicated in regulating mitochondrial calcium influx through voltage-dependent anion channels (VDACs) and mitochondrial calcium uniporter (MCU). Parkin ubiquitinates mitochondrial calcium uptake protein 1 (MICU1), a positive regulator of MCU, which is then rapidly degraded via the ubiquitin proteasomal system^6^. As Parkin also ubiquitinates phospholipase Cγ1 (PLCγ1), the upstream regulator of ER calcium release through the inositol 1,4,5-trisphosphate receptor (IP_3_R), Parkin deficiency leads to increased PLC activity and intracellular calcium levels^7^. Thus, PINK1 or Parkin deficiency affects the activity of their downstream targets which in turn modulates cytosolic and mitochondrial calcium concentrations. However, majority of these previously proposed targets localize to the mitochondrial inner membrane or plasma membrane, failing to correspond to the outer mitochondrial membrane localization of PINK1 and Parkin.

The CDGSH iron sulfur domain 1 (CISD1, also known as mitoNEET) protein contains a transmembrane domain and a CDGSH iron-sulfur (Fe-S) domain. The protein forms a homodimer in the mitochondrial outer membrane and localizes to the contact site between the mitochondrial outer membrane and the ER membrane, known as the mitochondrial-associated membrane (MAM)^10–14^. In addition, CISD1 is proposed to modulate the homeostasis of iron in the mitochondria^13,15,16^, and the mitochondria from CISD1-depleted mice exert dysregulated functions as indicated by elevated reactive oxygen species (ROS) production and decreased capacity to generate adenosine triphosphate (ATP)^17–19^. While these effects of CISD1 on mitochondrial functions support its potential role in the pathogenesis of several types of diseases including Parkinson’s disease, cancers, and diabetes^19–21^, the underlying molecular mechanisms remain elusive.

In the present study, we observe that IP_3_R activity is highly elevated in both PINK1 knockout (KO) and Parkin KO mammalian cells. A subsequent genetic screening of Parkin substrates in *Drosophila* revealed that CISD1/CISD is responsible for the increased IP_3_R activity and ER calcium release in PINK1 and Parkin KO cells and flies. The CDGSH domain of CISD1 is required for direct interaction between CISD1 and IP_3_R, and CISD1 KO cells have reduced IP_3_R activity, indicating that CISD1/CISD is crucial for IP_3_R activation. Treatment with pioglitazone, originally a type II diabetes medication that targets CISD1, inhibits IP_3_R-CISD1 binding and rescues the PD-related phenotypes of PINK1 and Parkin null *Drosophila* mutants. These results therefore demonstrate the robust relationship between PD pathogenesis and the intracellular calcium homeostasis controlled by the PINK1, Parkin, CISD1, and IP_3_R signaling axis.

## Results

### PINK1 and Parkin activity control ER calcium release by regulating IP_3_R activity

**Fig. 1:**
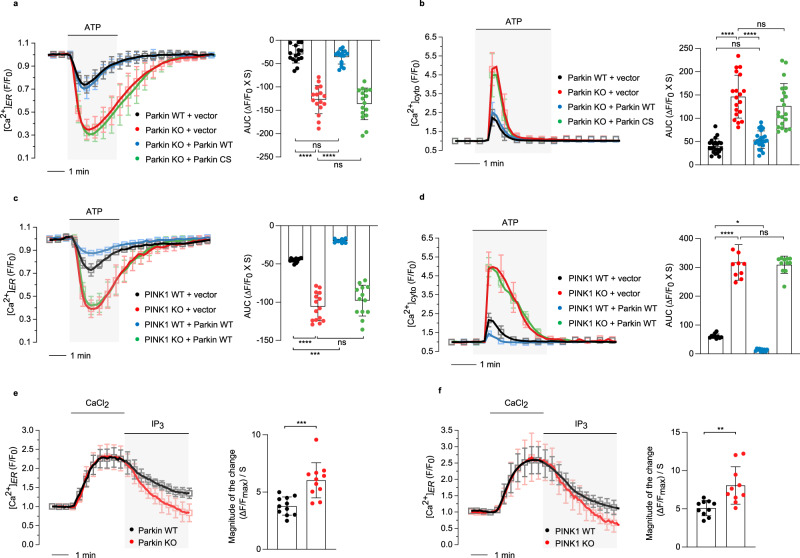
PINK1 and Parkin activity modulate ER calcium release through IP_3_R calcium channel. **a**, **b** Measurement of ER (**a**) and cytosolic (**b**) calcium modulation in WT (black) and Parkin KO (red) MEF cells. Similar experiments were also conducted for Parkin KO MEF cells expressing exogenous Parkin WT (blue), C431S (CS) mutant (green), or control empty vector (vector). 100 µM ATP was delivered to initiate IP_3_R-mediated calcium release. The right side bar graphs indicate the quantification of the normalized calcium traces using area-under-the-curve (AUC) of calcium release during ATP treatment. *n* = 162–231 cells. **c**, **d** Measurement of ER (**c**) and cytosolic (**d**) calcium modulation in WT (black) and PINK1 KO (red) MEF cells. Similar experiments were also conducted for PINK1 WT MEF cells expressing exogenous Parkin WT (blue) or control empty vector (vector), and PINK1 KO MEF cells expressing exogenous Parkin WT (green) or control empty vector (vector). The right side bar graphs indicate the quantification of the normalized calcium traces using AUC of calcium release during ATP treatment. *n* = 110–188 cells. **e**, **f** Measurement of IP_3_R activity. Cells were permeabilized for 100 sec with 20 µM β-escin in intracellular medium (ICM), and washed with ICM for 5 min. In all, 0.65 mM CaCl_2_ was then added to induce the influx of ER calcium. After a steady-state was achieved, 1 µM IP_3_ was introduced to evoke ER calcium release through IP_3_R. **e** Measurement of ER calcium release in WT (black, *n* = 72 cells) and Parkin KO (red, *n* = 68 cells) MEF cells. The bar graphs indicate the magnitude of the change during IP_3_ treatment. **f** Measurement of ER calcium release in WT (black, *n* = 65 cells) and PINK1 KO (red, *n* = 77 cells) MEF cells. Three independent experiments were conducted and were quantified (**a**–**f**). One-way analysis of variance (ANOVA) with Tukey’s multiple comparisons test was used (**a**–**d**), and two-tailed unpaired Student’s *t*-test was used (**e**, **f**). ****p < 0.0001. ***p < 0.001. **p < 0.01. *p < 0.05. ns represents not significant. Source data, the exact *p* values, and *n* number of each experiment are included within the Source Data file. All data are presented as mean ± SD.

To induce and measure ER calcium release in Parkin KO mouse embryonic fibroblast (MEF) cells, we treated cells with adenosine triphosphate (ATP), which triggers the production of inositol 1,4,5-trisphosphate (IP_3_) that binds to inositol 1,4,5-trisphosphate receptor (IP_3_R) to release calcium from the ER^22^, and used G-CEPIA1er as an ER-specific calcium indicator^23^. Unexpectedly, Parkin KO MEF cells showed highly increased ER calcium release upon ATP treatment compared to wild-type (WT) control cells (Fig. 1a). This increase was suppressed by the exogenous expression of Parkin WT but not by an inactive mutant of Parkin (Parkin C431S, indicated as Parkin CS; Fig. 1a), suggesting that the activity of the E3 ligase Parkin regulates ER calcium efflux. We also measured cytosolic calcium levels in response to ATP treatment using RCaMP1h as a calcium indicator. As a result of enhanced ER calcium release, Parkin KO MEF cells exhibited elevated cytosolic calcium levels, which were suppressed by expression of Parkin WT but not by Parkin CS (Fig. 1b).

We also tested whether PINK1, functionally upstream of Parkin, controls the release of ER calcium. Similar to Parkin KO cells, PINK1 KO MEF cells exhibited increased ER calcium release and cytosolic calcium levels compared to PINK1 WT cells, which were reduced by PINK1 WT expression (Supplementary Fig. 1a, b). However, expression of the PINK1 kinase-dead mutant (3KD; K219A/D362A/D384A) failed to rescue these ER and cytosolic calcium phenotypes (Supplementary Fig. 1a, b). In addition, we examined whether PINK1 is necessary for the regulation of ER calcium release by Parkin. We observed that upon expression of exogenous Parkin WT in PINK1 WT and KO cells, ER calcium release and cytosolic calcium levels decreased in PINK1 WT cells, but not in PINK1 KO cells (Fig. 1c, d). Expression of the Parkin C431S mutant, which lacks E3 ligase activity, in PINK1 WT or KO cells did not alter ER calcium release or cytosolic calcium levels (Supplementary Fig. 1c, d). These results indicated that the regulation of ER calcium release and cytosolic calcium levels is contingent upon the activity of Parkin and PINK1.

To understand how Parkin regulates ER calcium release, we first measured the activity of ER-localized calcium channels in Parkin KO MEF cells. Sarco/endoplasmic reticulum Ca^2+^-ATPase (SERCA) transports calcium from the cytosol into the ER, while IP_3_R is responsible for releasing ER calcium in response to IP_3_. We hypothesized that the loss-of-function of Parkin affects ER calcium flux through SERCA or IP_3_R and therefore measured the effect of the functional loss of Parkin on ER calcium uptake and release using G-CEPIA1er as an ER calcium indicator. To measure the respective activities of SERCA and IP_3_R, cells were first permeabilized with β-escin and then treated with calcium chloride to stimulate calcium influx through SERCA. The same cells were further treated with IP_3_ to induce ER calcium release through IP_3_R (Fig. 1e, f). While there were no significant differences in ER calcium uptake between Parkin WT and KO cells, we found that Parkin KO cells released significantly more ER calcium than WT cells by IP_3_ treatment (Fig. 1e). PINK1 KO cells also showed unchanged ER calcium uptake and increased ER calcium release compared to WT cells (Fig. 1f). In line with these findings, PINK1- and Parkin-deficient *Drosophila* mutants also exhibited increased IP_3_R activity when treated with IP_3_ compared to *w*^*1118*^ control flies (Supplementary Fig. 1e, f). Overall, these results suggested that loss of PINK1 and Parkin selectively increases IP_3_R activity.

### Parkin regulates IP_3_R activity through CISD1 and its *Drosophila* homolog CISD

**Fig. 2:**
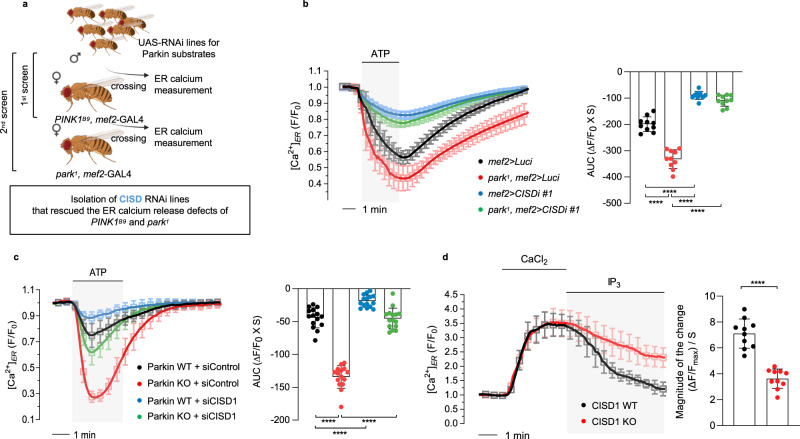
CISD1/CISD mediates Parkin-dependent regulation of ER calcium release. **a** Genetic scheme to screen new genes that alleviate defective ER calcium release of PINK1 (*PINK1*^*B9*^) and Parkin (*park*^*1*^) null mutants. To measure ER calcium release in *Drosophila* larval muscle, *mef2*-GAL4 were used to express ERGCaMP6-210, an ER calcium indicator. After dissection of the third instar larvae, fluorescence images of larval muscle were acquired by confocal microscope with a perfusion system. Some elements of the figure were created using images from BioRender.com. **b** Measurement of ER calcium release for control (*mef2>Luci*, black) and Parkin null mutants (red), CISD KD flies (blue), and Parkin null mutants expressing CISD RNAi (green). Here, we used *CISDi #1* flies (#33925 from the Vienna Drosophila Resource Center) for CISD KD. 5 mM ATP was delivered to initiate ER calcium release. The right side bar graphs indicate the quantification of the normalized calcium traces using AUC of calcium release during ATP treatment. *n* = 10 flies. **c** Measurement of ER calcium modulations in WT (black) and Parkin KO (red) MEF cells. Similar experiments were also conducted when WT (blue) and Parkin KO (green) MEF cells were expressing *CISD1* siRNA (siCISD1) or control siRNA (siControl). The right side bar graphs indicate the quantification of the normalized calcium traces using AUC of calcium release during ATP treatment. *n* = 158–195 cells. **d** Measurement of ER calcium release through IP_3_R in WT (black, *n* = 72 cells) and CISD1 KO (red, *n* = 79 cells.) MEF cells. The bar graphs indicate the magnitude of the change during IP_3_ treatment. Three independent experiments were conducted and were quantified (**b**–**d**). One-way ANOVA with Tukey’s multiple comparisons test was used (**b**, **c**), and two-tailed unpaired Student’s *t* test was used (**d**). ****p < 0.0001. Source data, the exact *p* values, and *n* number of each experiment are included within the Source Data file. All data are presented as mean ± SD.

To study how PINK1 and Parkin regulate IP_3_R activity, we performed a small-scale fly genetic screening (ref. ^24^; Fig. 2a), looking for changes in ER calcium release in PINK1 and Parkin fly mutants after knocking down 32 candidate proteins that are known substrates of Parkin and also localize to ER or mitochondrial membranes (Supplementary Table 1). We used flies expressing ERGCaMP6-210, an ER calcium indicator, and crossed them with PINK1 or Parkin null flies in addition to flies expressing candidate RNAi to measure ER calcium levels. The candidate RNAi lines were expressed using the *mef2*-GAL4 driver, which results in muscle-specific expression in *Drosophila*. Among the 32 candidate lines, 14 RNAi lines under the *mef2*-GAL4 driver were lethal so their ER calcium levels could not be measured (Supplementary Fig. 2a). Through our genetic calcium imaging screening, we successfully discovered that knockdown (KD) of CISD (also called as Dosmit^25^), the *Drosophila* homolog of human CISD1, suppressed the increased ER calcium release in PINK1- and Parkin-deficient flies (Fig. 2a, b and Supplementary Fig. 2b, c). To confirm whether CISD KD specifically suppresses the increased ER calcium release and cytosolic calcium levels in PINK1 or Parkin null flies, we used two different lines of CISD RNAi (#1: 33925 and #2: 104501 from the Vienna Drosophila Resource Center) which displayed decreased endogenous CISD protein levels compared to *hs*-GAL4 (Supplementary Fig. 3i–k) and *mef2*-GAL4 control flies (Supplementary Fig. 3h). In addition, to ensure that the transgene copy number for all fruit flies tested was equal when observing their phenotypes, we added the UAS-DsRed transgene into genetic crosses as designated in the figures. Consistent with the initial screening results, these two CISD RNAi fly lines showed decreased ER calcium release and cytosolic calcium levels compared to *mef2*-GAL4 control flies (Fig. 2b and Supplementary Figs. 4a–c and 5a–d). Additionally, increased ER calcium release and cytosolic calcium levels in PINK1- and Parkin-deficient flies were decreased when crossed with either CISD RNAi fly line (Fig. 2b, and Supplementary Figs. 4a–c and 5a–d). To further validate our findings in *Drosophila*, we generated CISD KO flies using the clustered regularly interspaced short palindromic repeats (CRISPR)-Cas9 system (Supplementary Fig. 3g, l), and measured ER calcium release and cytosolic calcium levels in *Drosophila*. CISD KO flies showed decreased ER calcium release and cytosolic calcium levels when treated with ATP compared to control flies (Supplementary Fig. 6a–d). The higher ER calcium release and cytosolic calcium levels observed in PINK1 and Parkin null flies were suppressed in a CISD-deficient background (Supplementary Fig. 6a–d).

We validated our results in the mammalian cell system, where the increased ER calcium release in PINK1 and Parkin KO MEF cells was suppressed by CISD1 siRNA expression (Supplementary Fig. 7a and Fig. 2c). In addition, higher cytosolic calcium levels were observed in PINK1 and Parkin KO MEF cells, all of which were suppressed with CISD1 KD (Supplementary Fig. 7b, c). We used immunoblot analyses to confirm the efficacy of CISD1 KD in MEF cells, and found that endogenous CISD1 protein levels were decreased in PINK1 and Parkin WT or KO MEF cells transfected with CISD1 siRNA, compared to cells transfected with control siRNA (Supplementary Fig. 3d, e). To confirm whether CISD1 KD in PINK1 and Parkin KO MEF cells suppresses ER calcium release levels by altering IP_3_R activity, we generated CISD1 KO MEF cells using the CRISPR-Cas9 system (Supplementary Fig. 3f) and measured ER calcium channel activity. As expected, permeabilized CISD1 KO MEF cells showed decreased ER calcium release when treated with IP_3_, indicating the reduction of IP_3_R activity (Fig. 2d). Also, in response to ATP treatment, CISD1 KO MEF cells exhibited decreased ER calcium release and cytosolic calcium levels compared to WT cells (See below). To further validate our findings in *Drosophila*, we measured IP_3_R activity following IP_3_ treatment and found that CISD deficiency led to decreased IP_3_R activity compared to *w*^*1118*^ control flies (Supplementray Fig. 6e). These results strongly supported that mammalian CISD1 and its *Drosophila* homolog CISD stimulate IP_3_R calcium channel activity.

### CISD1/CISD controls basal cytosolic calcium levels through regulation of IP_3_R activity

**Fig. 3:**
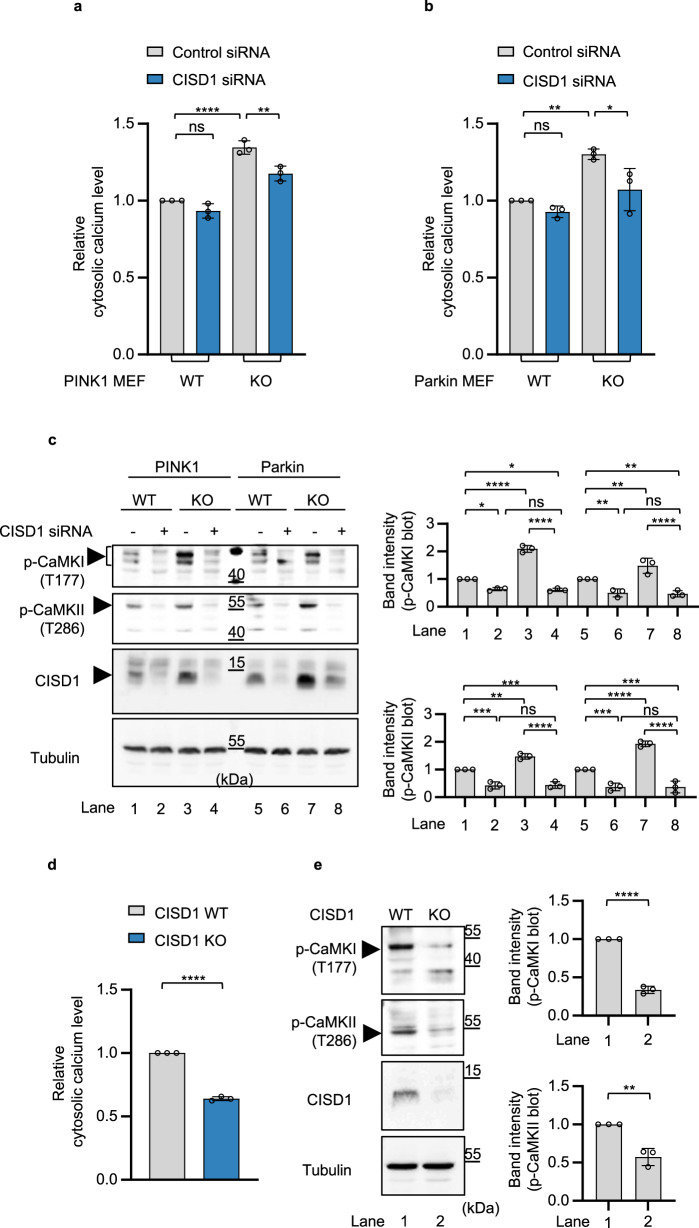
CISD1 controls basal cytosolic calcium levels. **a**, **b** Measurement of basal cytosolic calcium levels using FACS analysis. We stained PINK1 WT and KO (**a**) or Parkin WT and KO (**b**) MEF cells with Fluo-3 dye, and then measured the intensity of Fluo-3 fluorescence by FACS analysis. The cells were transfected with either control (gray) or CISD1 siRNA (blue). Bar graphs indicate relative basal calcium levels normalized to basal calcium levels of PINK1 WT (**a**) or Parkin WT (**b**) MEF cells. Three independent experiments were conducted and were quantified. Each sample was analyzed for 20,000 events using FACS. **c** Immunoblot analyses of cytosolic calcium signaling in PINK1 and Parkin WT or KO MEF cells when transfected with control (−) or CISD1 siRNA (+). Three independent experiments were conducted and the immunoblot band intensity for the phosphorylation of CaMKI and CaMKII were quantified (bar graphs in right). **d** Measurement of basal cytosolic calcium levels using FACS analysis in CISD1 WT (gray) or KO (blue) MEF cells. Bar graphs indicate relative basal calcium levels normalized to basal calcium levels of CISD1 WT MEF cells. Three independent experiments were conducted and were quantified. Each sample was analyzed for 20,000 events using FACS. **e** Immunoblot analyses of cytosolic calcium signaling in CISD1 WT or KO MEF cells. Three independent experiments were conducted and the immunoblot band intensity for the phosphorylation of CaMKI and CaMKII was quantitated (bar graphs in right). One-way ANOVA with Tukey’s multiple comparisons test was used (**a–c**), and two-tailed unpaired Student’s *t* test was used (**d**, **e**). ****p < 0.0001. ***p < 0.001. **p < 0.01. *p < 0.05. ns represents not significant. Source data and the exact *p* values are included within the Source Data file. All data are presented as mean ± SD.

We used Fluo-3 dye to measure basal intracellular calcium levels in cells with regulated CISD1 protein expression. Fluo-3 is a fluorescent indicator of intracellular calcium used to measure calcium inside living cells, and exhibits an increase in signal intensity upon binding with calcium^26,27^. We transfected CISD1 siRNA in PINK1 or Parkin WT and KO MEF cells and stained the cells with Fluo-3. The intensity of fluorescence was then measured in stained cells using fluorescence-activated cell sorting (FACS) analysis. PINK1 or Parkin KO cells demonstrated increased Fluo-3 fluorescence compared to PINK1 or Parkin WT cells (Fig. 3a, b). However, the increased intensity of Fluo-3 fluorescence in PINK1 or Parkin KO cells was subsequently reduced upon CISD1 siRNA expression (Fig. 3a, b). These results demonstrated that loss of PINK1 or Parkin increases basal intracellular calcium levels, whereas, CISD1 KD reverses this effect, resulting in a decrease in basal intracellular calcium levels. We performed additional immunoblot analyses to detect cytosolic calcium signaling, such as phosphorylation of calcium/calmodulin-dependent protein kinase I (CaMKI) and calcium/calmodulin-dependent protein kinase II (CaMKII). Previous studies have reported that CaMKI and CaMKII phosphorylation are increased when cytosolic calcium levels are elevated in cells^28,29^. We found that phosphorylation of CaMKI and CaMKII was increased in PINK1 or Parkin KO cells, but that these increased cytosolic calcium signals in PINK1 or Parkin KO cells were suppressed upon CISD1 KD (Fig. 3c). Additionally, in Parkin or PINK1 WT cells, we found that cytosolic calcium signals were reduced upon CISD1 KD compared to control siRNA expression (Fig. 3c). When we measured basal intracellular calcium levels using FACS analysis in CISD1 WT or KO MEF cells, CISD1 KO cells showed reduced basal cytosolic calcium levels compared to CISD1 WT cells (Fig. 3d). CISD1 KO cells also exhibited decreased cytosolic calcium signaling including phosphorylation of CaMKI and CaMKII (Fig. 3e).

To further validate the basal ER calcium changes in *Drosophila*, we expressed the ER calcium indicator ERGCaMP6-210 using the *mef2*-GAL4 driver, and measured the fluorescence intensity of ERGCaMP6-210 in dissected wandering third instar larvae. We implemented a negative control, the SERCA inhibitor thapsigargin (TG), and a positive control, the IP_3_R inhibitor 2-aminoethyl diphenylborinate (2-APB), which are known to modulate basal ER calcium. Our results demonstrated a dose-dependent decrease in basal ER calcium levels following TG treatment as well as a dose-dependent increase after treatment with 2-APB (Supplementary Fig. 8a, b). We then measured the fluorescence intensity of ERGCaMP6-210 in PINK1 or Parkin KO flies that additionally had reduced CISD, through expression of one of the two CISD RNAi lines, or complete deficiency of CISD through CISD KO. Our findings revealed that the fluorescence intensity of ERGCaMP6-210 was decreased in PINK1 KO and Parkin KO flies compared to *mef2*-GAL4 control flies (Supplementary Fig. 8c, d). In contrast, the fluorescence intensity of ERGCaMP6-210 was increased in CISD KO and the two CISD KD flies (Supplementary Fig. 8c, d). These data demonstrated that basal ER calcium levels were reduced in PINK1 and Parkin KO flies in comparison with control flies. The reduced basal ER calcium levels in PINK1 KO and Parkin KO flies were increased when these flies were crossed with either CISD RNAi line or in CISD KO background (Supplementary Fig. 8c, d). To quantify basal cytosolic calcium levels, we used the GCaMP5G cytosolic calcium indicator. Consistent with the ER calcium results, PINK1 and Parkin KO showed increased basal cytosolic calcium levels compared to control flies (Supplementary Fig. 8e, f). The increased basal cytosolic calcium levels in PINK1 and Parkin KO flies were reduced by decreasing CISD expression through crossing RNAi lines or complete deletion in CISD KO background (Supplementary Fig. 8e, f). These results demonstrated that CISD1/CISD controls basal intracellular calcium levels in both mammalian cells and *Drosophila*.

### CISD1 is a substrate of the E3 ligase Parkin

**Fig. 4:**
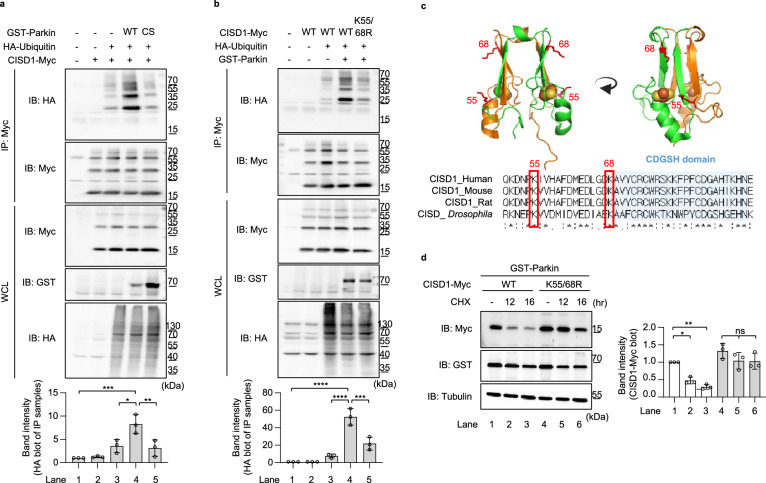
CISD1 is destabilized by Parkin-dependent ubiquitination. **a** Ubiquitination levels of CISD1 in HEK293T cells expressing Parkin WT or C431S (CS). The cells were treated with 20 µM MG132, 1 µM antimycin, and 10 µM oligomycin for 4 h and lysed for anti-Myc immunoprecipitation to compare the level of CISD1 ubiquitination by immunoblot analyses. Three independent experiments were conducted and the immunoblot band intensity for ubiquitinated CISD1 were quantitated (bar graphs in bottom). **b** Ubiquitination levels of CISD1 WT and K55R/K68R (K55/68R) double mutant in HEK293T cells expressing Parkin WT. The cells were treated with 20 µM MG132, 1 µM antimycin, and 10 µM oligomycin for 4 h and lysed for anti-Myc immunoprecipitation to compare the level of CISD1 ubiquitination by immunoblot analyses. Three independent experiments were conducted and the immunoblot band intensity for ubiquitinated CISD1 were quantitated (bar graphs in bottom). **c** Aligned CISD1 protein sequences of human (Q9NZ45.1), mouse (NP_598768.1), rat (B0K020.1), and *Drosophila* (Q9VAM6.1) with indication of the conserved lysine residues in red boxes. Three-dimensional human CISD1 protein structures, RCSB PDB-2QD0^11^, show K55 and K68 ubiquitination sites using PyMOL v2.4.0. **d** Immunoblot analyses of CISD1 protein showed its reduced stability by Parkin-dependent K55 and K68 ubiquitination in HEK293T cells. The cells were treated with 100 µg/µl cycloheximide (CHX) for 12 or 16 h and analyzed the level of CISD1 protein. Three independent experiments were conducted and the immunoblot band intensity for CISD1 were quantitated (bar graphs in right). One-way ANOVA with Tukey’s multiple comparisons test was used (**a**, **b**, **d**). ****p < 0.0001. ***p < 0.001. **p < 0.01. *p < 0.05. ns represents not significant. Source data and the exact *p* values are included within the Source Data file. All data are presented as mean ± SD.

To confirm whether the CISD1 protein is a direct target of Parkin, we performed a ubiquitination assay for CISD1 with Parkin WT and the CS mutant. As expected, CISD1 was ubiquitinated by Parkin WT in cells treated with antimycin and oligomycin (Fig. 4a). However, the catalytically inactivated Parkin CS mutant did not ubiquitinate CISD1 (Fig. 4a). We then searched for the sites of CISD1 that are ubiquitinated by Parkin. CISD1 has well-conserved lysine residues among various species (Fig. 4c), of which we found that the CISD1 K55/68 R double mutant was barely ubiquitinated by Parkin (Fig. 4b). To investigate the effect of CISD1 ubiquitination by Parkin, we treated Parkin-transfected cells with cycloheximide (CHX) and observed the protein stability of CISD1 WT and the K55/68R double mutant (Fig. 4d). Interestingly, protein levels of the CISD1 K55/68 R double mutant were highly stabilized compared to those of CISD1 WT (Fig. 4d). Furthermore, endogenous CISD1/CISD protein levels in PINK1 and Parkin KO MEF cells as well as PINK1 and Parkin null flies were elevated (Supplementary Fig. 3a–c). These results consistently demonstrate that Parkin ubiquitinates CISD1 protein and promotes its degradation.

### Loss of CISD rescues PINK1 and Parkin deficiency through regulation of IP_3_R activity

**Fig. 5:**
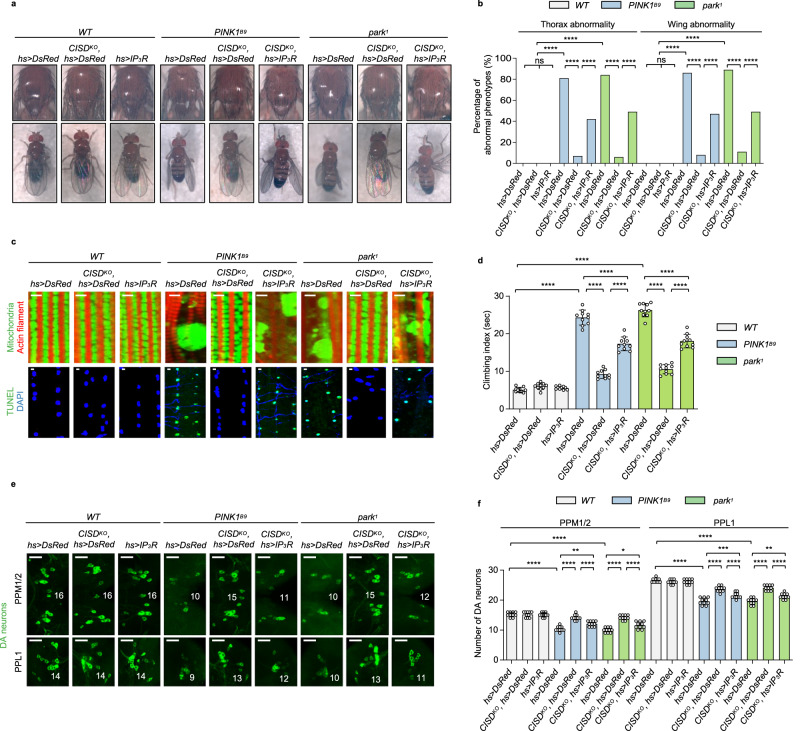
Loss of CISD rescues the PD-related phenotypes of PINK1 and Parkin mutants through regulating IP_3_R. **a**, **b** Images of *Drosophila* thoracic (top panels) and wing posture (bottom panels) phenotypes (**a**) and percentages of the flies having abnormal phenotypes (**b**). *hs* > *IP*_*3*_*R* indicates heat shock-induced overexpression of the *Drosophila* IP_3_R gene, *itpr*. *CISD*^*KO*^ indicates the KO genetic background of CISD in *Drosophila*. **c** Immunofluorescence images of the adult flight muscles (top). Green (mitochondria) and red (actin filament). *n* = 10. Scale bars, 5 µm. TUNEL assays of the adult flight muscles (bottom). Green (TUNEL) and blue (DAPI). *n* = 10. Scale bar, 5 µm. **d** Measurement of the climbing ability in the adult flies with indicated genotypes. *n* = 10. **e**, **f** Immunofluorescence images (**e**) and number (**f**) of DA neurons in PPM1/2 (top), and PPL1 (bottom) regions of adult fly brains with indicated genotypes. Green (DA neurons). Images of PPL1 regions were obtained from one of the left or right side of the PPL1 regions. Numbers of the DA neurons in the PPL1 regions were counted from both hemispheres*. n* = 10. Scale bars, 20 µm. For statistical analysis, one-sided Chi-square test was used (**b**). One-way ANOVA with Tukey’s multiple comparisons test was used (**d**, **f**). ****p < 0.0001. ***p < 0.001. **p < 0.01. *p < 0.05. ns represents not significant. Source data and the exact *p* values are included within the Source Data file. All data are presented as mean ± SD.

Our previous studies have shown that PINK1- and Parkin-deficient flies exhibit abnormal mitochondrial morphology, defects in wing postures, and increased thoracic apoptotic signals^30,31^. Also, flies lacking PINK1 or Parkin show a decreased climbing ability and loss of DA neurons in protocerebral posterior medial 1/2 (PPM1/2) and posterior protocerebrum lateral 1 (PPL1)^30–32^. Excitingly, these phenotypes of PINK1 and Parkin null flies – including crushed thoraces, abnormal wing postures, abnormal mitochondrial morphology in thoraces, increased apoptotic responses in the muscle, impaired climbing ability, and reduced number of DA neurons in the PPM1/2 and PPL1 regions of the adult brain – were fully rescued with CISD KD or KO (Fig. 5a–f and Supplementary Figs. 9a–h, 10a–f, and 11a, b), demonstrating that the suppression of CISD rescues the PD-related pathogenesis induced by PINK1 or Parkin deficiency. Such rescuing effects of CISD KD and KO in PINK1 and Parkin null flies were blocked by simultaneous expression of exogenous CISD WT (Supplementary Figs. 9a–h, 10a–f, and 11a, b).

To clarify whether the rescuing effects of CISD KD and KO are due to reduced IP_3_R activity, we observed PD-related phenotypes in PINK1 or Parkin null flies co-expressing CISD RNAi and exogenous *Drosophila* IP_3_R. While CISD KD and KO in PINK1 and Parkin null flies completely rescued PD-related phenotypes (Fig. 5a–f and Supplementary Figs. 9a–h, 10a–f, and 11a, b), simultaneous expression of IP_3_R resulted in recurrence of PD-related phenotypes, indicating that the rescuing effects of CISD KD and KO were strongly diminished (Fig. 5a–f and Supplementary Figs. 9a–h and 11a, b). Furthermore, we examined whether reduced IP_3_R activity (Supplementary Fig. 12a) could rescue PD-related phenotypes of PINK1 and Parkin null flies. Expectedly, IP_3_R KD partially rescued all PD-related phenotypes of PINK1 and Parkin null flies including crushed thoraces, abnormal wing postures, abnormal mitochondrial morphology, increased apoptotic signals, defective climbing ability, and reduced number of DA neurons (Supplementary Fig. 12b–i). Taken together, we suggested that loss of CISD decreases IP_3_R activity, which thus normalizes intracellular calcium homeostasis and rescues PD-related pathogenesis in PINK1 or Parkin null flies.

### CISD1 controls IP_3_R activity through direct interaction with IP_3_R

**Fig. 6:**
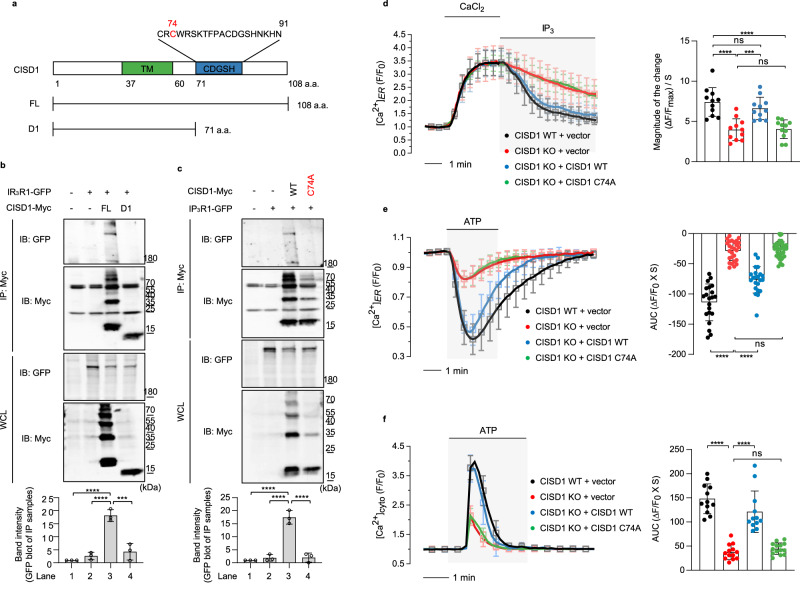
CISD1 directly binds to IP_3_R and increases its calcium channel activity. **a** The domain architecture of human CISD1. The transmembrane domain (TM) and the CDGSH domain are indicated. The FL represents the full-length protein of human CISD1. We generated the D1 construct which lacks the CDGSH domain. **b**, **c** HEK293T cells were transfected as indicated and cell lysates were subjected to anti-Myc immunoprecipitation followed by immunoblot analysis. Three independent experiments were repeated and the band intensity for the interaction between CISD1 and IP_3_R1 shown by co-immunoprecipitation experiments was displayed in bar graphs (bottom). **d** Measurement of the IP_3_R activity of WT (black, *n* = 63) and CISD1 KO (red, *n* = 68) cells when transfected with exogenous empty vector (vector, black and red), CISD1 WT (blue, *n* = 70), and CISD1 C74A mutant (green, *n* = 74). The bar graphs indicate the magnitude of the change during IP_3_ treatment. **e**, **f** Measurement of ER (**e**) and cytosolic (**f**) calcium modulation in WT (black) and CISD1 KO (red) MEF cells with empty vector (vector). Similar experiments were also conducted for CISD1 KO MEF cells expressing exogenous CISD1 WT (blue) or C74A mutant (green). In all, 100 µM ATP was delivered to initiate IP_3_R-mediated calcium release. The right side bar graphs indicate the quantification of the normalized calcium traces using AUC of calcium release during ATP treatment. *n* = 114–162 cells. One-way ANOVA with Tukey’s multiple comparisons test was used (**b**–**f**). ****p < 0.0001. ***p < 0.001. ns represents not significant. Source data, the exact *p* values, and *n* number of each experiment are included within the Source Data file. All data were presented as mean ± SD.

It has been previously reported that IP_3_R activity can be regulated by several proteins, such as sigma-1 receptor^33^, B-cell lymphoma-2 (BCL-2)^34^, and BRCA1-assicated protein 1 (BAP1)^35^. These proteins directly interact with IP_3_R to regulate its activity. To verify whether the CISD1 protein directly binds to IP_3_R, we performed co-immunoprecipitation experiments for CISD1 and IP_3_R1 proteins. We found that bovine IP_3_R1 directly interacts with CISD1 and further narrowed down that the CDGSH domain of CISD1 is responsible for this interaction (Fig. 6a, b). To identify the critical amino acid residue of CISD1 that interacts with IP_3_R1, we generated various mutations in the CDGSH domain. We found that the C74A mutant form of CISD1 did not bind to IP_3_R1 (Fig. 6c), suggesting that the cysteine 74 of the CISD1 protein is critical for the interaction between CISD1 and IP_3_R1. Moreover, the decreased IP_3_R activity in CISD1 KO MEF cells was rescued by the expression of CISD1 WT but not by the CISD1 C74A mutant (Fig. 6d). CISD1 WT expression also increased ER calcium release and cytosolic calcium levels, whereas expression of the CISD1 C74A mutant failed to do so (Fig. 6e, f). These results support that the direct interaction between CISD1 and IP_3_R is necessary for maintaining IP_3_R activity.

### Pioglitazone rescues the PD-related phenotypes induced by PINK1 or Parkin deficiency

**Fig. 7:**
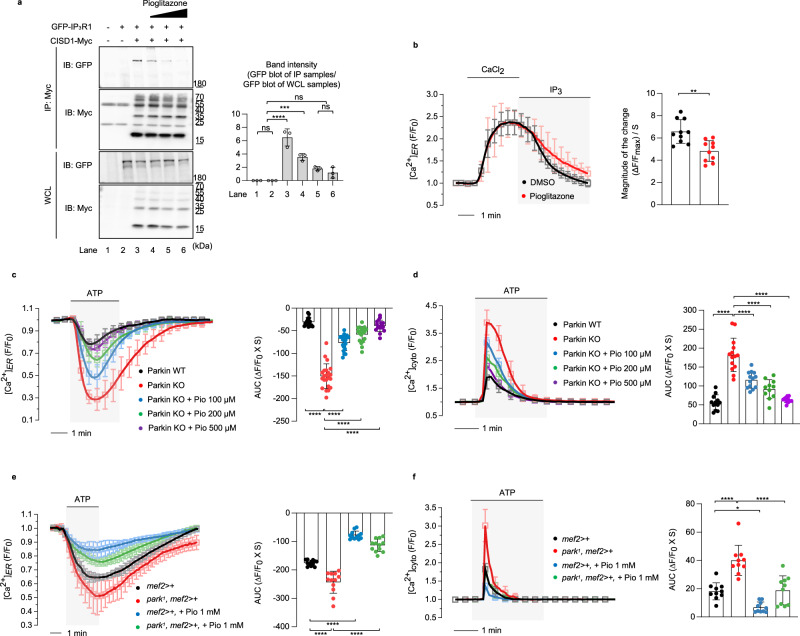
Pioglitazone, a CISD1 inhibitor, inhibits IP_3_R activity and restores the defective ER calcium release caused by Parkin deficiency. **a** HEK293T cells were transfected as indicated and treated with pioglitazone. Anti-Myc immunoprecipitates were analyzed for the association of IP_3_R1 using immunoblot analyses. The binding of IP_3_R and CISD1 was inhibited by a dose-dependent treatment of pioglitazone (100, 200, and 500 µM) for 30 min. Three independent experiments were done to quantitate the interaction between CISD1 and IP_3_R1 (bar graphs in right). **b** Measurement of the IP_3_R activity in HEK293 cells treated with 200 µM pioglitazone. The magnitude of the change during IP_3_ treatment was shown in bar graphs (right). *n* = 75–81 cells. **c**, **d** Analyses of ER and cytosol calcium modulations in Parkin KO cells when treated with pioglitazone. Measurement of ER (**c**) and cytosol (**d**) calcium modulations in WT (black) and Parkin KO (red) cells when treated with indicated concentrations of pioglitazone (blue, green, and purple). The right side bar graphs indicate the quantification of the normalized calcium traces using AUC of calcium release during ATP treatment. *n* = 85–127 cells. **e**, **f** Measurement of ER (**e**) and cytosol (**f**) calcium modulations in control (*mef2*>*+*, black) and Parkin null mutant flies (*park*^*1*^, red) when treated without or with 1 mM pioglitazone (blue and green). The right side bar graphs indicate the quantification of the normalized calcium traces using AUC of calcium release during ATP treatment. *n* = 12 flies (**e**). *n* = 10 flies (**f**). Two-tailed unpaired Student’s *t* test was used (**b**), and one-way ANOVA with Tukey’s multiple comparisons test was used (**a** and **c**–**f**). ****p < 0.0001. ***p < 0.001. **p < 0.01. *p < 0.05. ns represents not significant. Source data, the exact *p* values, and *n* number of each experiment are included within the Source Data file. All data are presented as mean ± SD.

The antidiabetic drug pioglitazone has been reported to bind to CISD proteins and inhibit the [2Fe-2S] cluster transfer upon binding^14,36^. To determine whether pioglitazone affects the interaction between IP_3_R and CISD proteins, we performed co-immunoprecipitation experiments for CISD1 and IP_3_R1 with pioglitazone treatment. Interestingly, the addition of pioglitazone reduced the binding between IP_3_R1 and CISD1 in a dose-dependent manner (Fig. 7a). We also found that pioglitazone treatment reduced IP_3_R activity (Fig. 7b). Then, we measured ER calcium release in PINK1 and Parkin KO cells with pioglitazone treatment and found that, as expected, the increased ER calcium release in PINK1 and Parkin KO MEF cells was suppressed with pioglitazone (Supplementary Fig. 13a and Fig. 7c). ER calcium release was further diminished as pioglitazone dosage was increased in PINK1 and Parkin KO MEF cells (Supplementary Fig. 13a and Fig. 7c). The increased cytosolic calcium levels in PINK1 and Parkin KO cells were decreased by pioglitazone in a dose-dependent manner as well (Supplementary Fig. 13b and Fig. 7d). PINK1 and Parkin WT cells also displayed similar trends, where ER calcium release were decreased by pioglitazone in a dose-dependent manner (Supplementary Fig. 13c, d). Consistent with these results, PINK1 and Parkin null flies showed increased ER calcium release and cytosolic calcium levels compared to WT flies, which were rescued by pioglitazone treatment (Supplementary Fig. 13e, f and Fig. 7e, f), demonstrating again that CISD1/CISD is a critical mediator of PINK1 and Parkin to control IP_3_R activity.

**Fig. 8:**
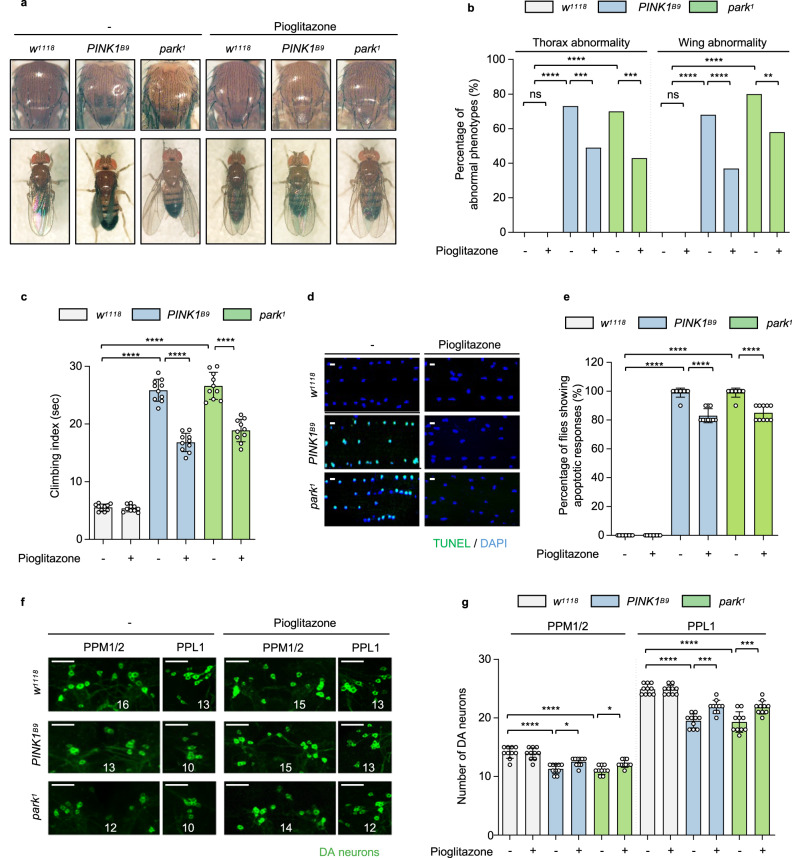
Pioglitazone rescues the PD-related phenotypes of PINK1 or Parkin deficiency. **a**–**g** Fruit fly eggs were transferred to a medium containing 1 mM pioglitazone (+) or DMSO vehicle (−), and we performed the following experiments on 3- and 30-day-old adult flies. **a**, **b** Images of *Drosophila* thoracic (top panels) and wing posture (bottom panels) phenotypes (**a**) and percentages of the flies having abnormal phenotypes (**b**). The flies in right panels (Pioglitazone) were fed with 1 mM pioglitazone sucrose solution (+) and compared with controls fed with vehicle-containing sucrose solution (−). **c** Measurement of the climbing ability of the adult flies with indicated genotypes. + flies were fed with 1 mM pioglitazone sucrose solution. *n* = 10. **d**, **e** Images (**d**) and quantification (**e**) of the TUNEL assays of the adult flight muscles with indicated genotypes. + flies were fed with 1 mM pioglitazone sucrose solution. Green (TUNEL) and blue (DAPI). *n* = 10. Scale bars, 5 µm. **f**, **g** Immunofluorescence images (**f**) and number (**g**) of DA neurons in PPM1/2, and PPL1 regions of adult fly brains with indicated genotypes. Images of PPL1 regions were obtained from one of the left or right side of the PPL1 regions. Numbers of the DA neurons in the PPL1 regions were counted from both hemispheres. + flies were fed with 1 mM pioglitazone sucrose solution. Green (DA neurons). *n* = 10. Scale bars, 20 µm. For statistical analysis, one-sided Chi-square test was used (**b**). One-way ANOVA with Tukey’s multiple comparisons test was used (**c**, **e**, **g**). ****p < 0.0001. ***p < 0.001. **p < 0.01. *p < 0.05. ns represents not significant. Source data and the exact *p* values are included within the Source Data file. All data are presented as mean ± SD.

Finally, we investigated whether pioglitazone could cure PD-related pathogenesis by feeding pioglitazone to PINK1 and Parkin null flies. Interestingly, pioglitazone feeding markedly rescued the PD-related phenotypes found in PINK1 and Parkin null flies such as crushed thoraces, abnormal wing postures, decreased climbing ability, increased apoptotic signals in thoraces, and reduced number of DA neurons (Fig. 8a–g). Taken together, our data suggested that pioglitazone is an effective therapeutic to treat the PD pathogenesis caused by PINK1 and Parkin mutations.

## Discussion

**Fig. 9:**
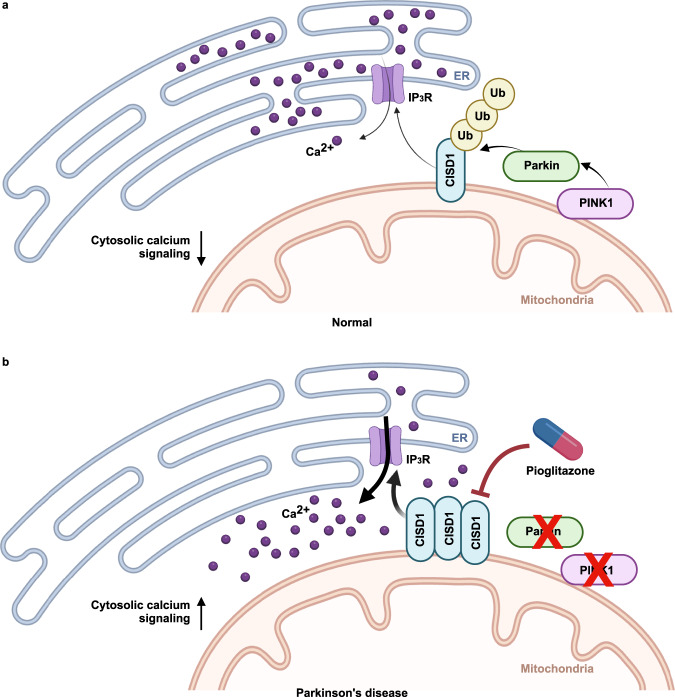
Loss of PINK1 or Parkin leads to dysregulation of ER and cytosolic calcium homeostasis. **a** In normal conditions, PINK1 activates the Parkin E3 ligase that results in ubiquitination and degradation of CISD1, located on the outer mitochondrial membrane. As CISD1 normally activates the ER calcium channel IP_3_R, inhibition of CISD1 by Parkin leads to suppressed IP_3_R activity and decreased cytosolic calcium levels. **b** However, in PD conditions induced by PINK1 or Parkin mutations, increased CISD1 protein levels cause elevated IP_3_R activity and cytosolic calcium levels. The inhibitor of CISD1 pioglitazone suppresses IP_3_R activity and restores calcium homeostasis, and therefore rescues the PD phenotypes caused by loss of PINK1 or Parkin. This figure was created with images from BioRender.com.

Our study provides a new insight into the mechanistic connection between the dysregulation of intracellular calcium homeostasis and PD pathogenesis induced by PINK1 or Parkin deficiency. PINK1 or Parkin KO mammalian cells exhibit increased IP_3_R activity, leading to increased ER calcium release and cytosolic calcium levels. We found that CISD1, a substrate of Parkin, directly controls IP_3_R activity and ER calcium release, indicating that PINK1, Parkin, CISD1, and IP_3_R all function in the same essential pathway that regulates ER and cytosolic calcium homeostasis (Fig. 9a). Loss of CISD or treatment with the CISD inhibitor pioglitazone restores the elevated ER calcium release in PINK1 and Parkin null flies and fully rescues their PD-related phenotypes. Taken together, the increased IP_3_R activity and ER calcium release caused by PINK1 and Parkin deficiency are key to PD pathogenesis, all of which can be rescued by suppression of CISD1 activity (Fig. 9b).

In humans, there are three isoforms of CISD, CISD1, CISD2 (also known as ERIS, Miner1, NAF-1, WFS2, and ZCD2), and CISD3 (also called as MiNT). Among the three isoforms, CISD1 and CISD2 contain a single CDGSH domain and a transmembrane domain that facilitates their anchoring to the outer membrane of mitochondria and the ER, respectively^37^. These two isoforms form homodimers within their respective organelles^37^. CISD3 functions as a monomer and contains two CDGSH domains. CISD3 localizes specifically to the mitochondrial matrix^38^. However, no isoforms exist in *Drosophila* CISD and this single protein shows sequence similarity with both human CISD1 and CISD2. We selected isoform CISD1 for our experiment, as CISD1 is a much better substrate for Parkin E3 ligase compared to CISD2^39–41^. Furthermore, it is well known that Parkin localizes to the mitochondria upon its activation and subsequently, ubiquitinates mitochondrial protein substrates^39–42^. When we observed subcellular localization of human CISD1/2 and *Drosophila* CISD proteins, human CISD1 and *Drosophila* CISD were localized in the mitochondria; however, human CISD2 was localized in the ER (Supplementray Fig. 14). Considering these points, we selected human CISD1 as the mammalian counterpart of *Drosophila* CISD and performed our experiments accordingly. Interestingly, Chang et al. previously demonstrated that CISD2 is required for BCL2 to suppress IP_3_R activity^43,44^. Thus, our study on the regulatory mechanism of IP_3_R activity through CISD1 is distinct from Chang et al.’s study on the regulation of IP_3_R activity by CISD2. Despite the structural and functional similarities between CISD1 and CISD2, the two proteins have distinct subcellular localizations and are involved in roles independent of one another, due to their interactions with different proteins. Overall, the results of our and Chang et al.’s study suggest that both CISD1 and CISD2 are modulators of IP_3_R activity, but they do so via their unique mechanisms that are distinctive of each other.

Previous studies reported that CISD1 is involved in iron homeostasis and the downregulation of CISD 1 causes iron accumulation and ROS production in mitochondria^16,18,45–48^. In light of these effects, we measured ROS levels in PINK1 and Parkin WT or KO mammalian cells and *Drosophila*, and confirmed an increase in ROS levels in PINK1 and Parkin KO MEF cells and *Drosophila* (Supplementary Fig. 15a–c). We also observed an increase in ROS levels in CISD KD and KO flies, compared to control flies (Supplementary Fig. 15b, c). Interestingly, CISD1/CISD KD or KO in PINK1 and Parkin KO cells and *Drosophila* resulted in similar ROS levels compared to PINK1 and Parkin KO cells and *Drosophila* (Supplementary Fig. 15a–c). Furthermore, the increased ROS levels in PINK1 or Parkin KO cells and *Drosophila* were not restored when CISD1/CISD was knocked down or deleted (Supplementary Fig. 15a–c). These results implicated that the rise in ROS levels induced by loss of CISD1/CISD is not directly involved in the rescue of PD phenotypes, which we observed in CISD1/CISD loss-of-function experiments.

The Fe-S binding capability of CISD1 may play a role in its interactions with IP_3_R. CISD1 has been reported to interact with several proteins, including CISD2, VDAC1, and transferrin receptor (TfR). CISD2^49^, VDAC1^15,50^, and TfR^51^ proteins have been shown to interact with each other, and this interaction has been implicated in the regulation of iron homeostasis, redox signaling, and Fe-S cluster synthesis in the mitochondria^15,49–51^. However, whether the Fe-S binding motif of CISD1 plays an essential role in protein-protein interactions is unclear. We tested whether the functions of CISD1 related to Fe-S binding are important to regulate IP_3_R activity and identified that the cysteine 74 residue in the Fe-S binding motif (in the CDGSH domain) of CISD1 is critical for the interaction with IP_3_R1 (Fig. 6c and Supplementary Fig. 16a–c). However, we also confirmed that the Fe-S binding motif of CISD1 binds with IP_3_R despite C72A substitution and CDGSH pentapeptide deletion mutations (Supplementary Fig. 16a–c). Thus, we postulate that the structural change in the Fe-S binding motif of CISD1 does not affect the binding between CISD1 and IP_3_R and that pioglitazone reduces the binding of CISD1 with IP_3_R regardless of the stability of Fe-S binding. Altogether, the Fe-S binding ability of CISD1 is not directly related to regulating IP_3_R activity.

Flies with either CISD RNAi or CISD KO exhibited lower ER calcium release and cytosolic calcium levels compared to *mef2*-GAL4 control flies (Fig. 2b and supplementary Figs. 4, 5, and 6a–d). Upon crossing with CISD RNAi or CISD KO flies, PINK1 or Parkin null flies displayed a greater reduction in ER calcium release and cytosolic calcium levels than *mef2-*GAL4 control flies (Fig. 2b and supplementary Fig. 4, 5, and 6a–d). This observation can be explained by the varying amounts of endogenous CISD in the flies. Notably, CISD RNAi flies exhibited significantly lower endogenous CISD amounts compared to the control flies (Supplementary Fig. 3h–k), while PINK1 or Parkin KO flies presented higher levels (Supplementary Fig. 3a, g–h, l). The elevated amount of endogenous CISD protein in PINK1 or Parkin KO flies contributes to the increased ER calcium release observed in these flies, while the reduced endogenous CISD protein in CISD RNAi or CISD KO flies results in a more significant decrease in ER calcium release compared to the control flies. Moreover, flies resulting from the crossing of PINK1/Parkin KO with CISD RNAi/CISD KO demonstrated lower endogenous CISD levels compared to the control flies, leading to a larger reduction in ER calcium release or cytosolic calcium levels. Collectively, these findings proposed that ER calcium release and cytosolic calcium levels are modulated proportionally to the amount of endogenous CISD protein present.

Defects in ER calcium homeostasis can also have profound effects on other organelles through physical contact sites, including the ER-mitochondria interconnections known as MAMs^52–57^. MAMs are enriched with the MCU complex in the inner mitochondrial membrane and IP_3_R on the ER membrane. MCU and IP_3_R are coupled via the glucose-regulated protein 75 (Grp75), which links IP_3_R to the VDAC1 on the outer mitochondrial membrane, establishing connections that allow calcium exchange between the ER and mitochondria^58^. Interestingly, our previous studies show that inhibition of MCU or VDAC1 partially rescues the PD phenotypes of PINK1- and Parkin-deficient flies, suggesting that the disruption of MAMs may alleviate PD pathogenesis^59^. Previous studies also report that the level of MAM contacts was increased in cultured human fibroblasts from PD patients carrying PINK1 or Parkin pathogenic mutations and PINK1 and Parkin null mutant flies^60^. In addition, our present results demonstrate that CISD1 directly binds to and regulates IP_3_R activity, and CISD1 is localized at MAMs and the mitochondrial outer membrane^12^. These data therefore suggest that CISD1 and the PINK1-Parkin pathway are crucial for the formation and maintenance of MAM structure and ER-mitochondrial calcium transduction, which in turn are critical for mitochondria-related physiology and pathologic phenotypes including calcium-dependent metabolic changes, ROS production, mitophagy, mitochondrial permeability transition, and apoptosis.

Through extensive studies, we understand that loss of PINK1 or Parkin impairs mitophagy and that defective mitophagy is one of the potential contributing factors to the onset of PD^61–67^. Furthermore, a recent study has shown increased mitophagy in thoraces and neurons of CISD KO or KD *Drosophila*, and the reduced mitophagy in PINK1 or Parkin KO flies was alleviated by crossing them with CISD KO or KD flies^68^. In our study, we observed that loss of CISD1/CISD reduced the elevated cytosolic calcium levels observed in PINK1 or Parkin KO cells and *Drosophila*. Intracellular calcium signaling is an important factor in mitophagy regulation^69^. Nix, also known as BCL2 interacting protein 3 like (BNIP3L), exhibits biological activity at both the mitochondria and the ER^70^. At the mitochondria, Nix functions as a selective autophagy receptor, facilitating the recruitment of LC3B^71^. In muscle, during a mitophagy response, Nix promotes ER-dependent calcium signaling to activate the mitochondrial fission regulator dynamin-related protein 1 (DRP1), indicating the contribution of Nix to mitophagy^72^. During hypoxia, mitochondrial Lon protein promotes FUNDC1-ULK1-mediated mitophagy at the MAMs, which depends on its binding with mitochondrial Na^+^/Ca^2+^ exchanger (NCLX). This interaction stabilizes the FUNDC1-ULK1 at the MAMs and initiates the mitophagy by regulating calcium levels between the mitochondria and cytosol^73^. This process occurs independently of PINK1 and Parkin. Furthermore, other calcium-sensitive proteins and pathways may also contribute to PINK1-Parkin-independent mitophagy. For example, CaMKII-AMP-activated protein kinase (AMPK) pathway has been implicated in the regulation of mitophagy. Activation of AMPK by CaMKII can promote mitophagy by phosphorylating and activating proteins involved in autophagy initiation^74,75^. This suggests the possibility that mitophagy could be activated by the decreased cytosolic calcium levels in CISD1/CISD KO or KD cells and *Drosophila*. Collectively, the regulation of mitophagy by CISD1/CISD holds the potential to alleviate PD pathogenesis caused by loss of PINK1 or Parkin. However, further investigation is required to unravel the molecular mechanism underlying mitophagy regulation by CISD1 and its interplay with intracellular calcium signaling.

Although degeneration of DA neurons is known to occur in PD, how such selective neurodegeneration occurs remains unknown. Our results show that DA neuronal loss and locomotor impairments in PINK1 and Parkin KO flies can be rescued by adjusting ER calcium release, suggesting that ER and mitochondrial calcium dysregulation may cause selective DA neuronal death. Intracellular calcium signaling in DA neurons is extremely fine-tuned as it controls many cellular processes including gene transcription, membrane excitability, dopamine neurotransmitter secretion, and synaptic plasticity^76–81^. Furthermore, energy production in neurons is tightly regulated by ER and mitochondrial calcium^76,82^. DA neurons promote mitochondria calcium influx from the ER to stimulate OXPHOS and the production of ATP^79,83^. This bioenergetic control system is costly, as enhancing OXPHOS in the absence of strong ATP demand leads to mitochondrial hyperpolarization, retrograde electron flux through the electron transport chain, and increased production of ROS^84,85^. Therefore, continuous dysregulation of calcium homeostasis in DA neurons along with exposure to risk factors (i.e., aging, mitochondrial toxins, mutations) may selectively induce metabolic stress and mitochondrial damages, leaving DA neurons more vulnerable than other neuronal populations to death^86–89^.

While the importance of calcium regulation in PD pathogenesis has been recognized, previous trials of calcium-related drugs had failed to improve symptoms in PD patients^90,91^. Our study proposes that pioglitazone, a thiazolidinedione (TZD) and antidiabetic drug, can alleviate PD pathogenesis. Though previous clinical studies have reported mixed results on the effectiveness of pioglitazone against PD^92–94^, our results clearly demonstrate that feeding pioglitazone to flies rescues PD-related phenotypes induced by PINK1 or Parkin deficiency. In addition, pioglitazone treatment reverses the increased ER calcium release and cytosolic calcium levels in PINK1 and Parkin KO MEF cells. We thus establish that pioglitazone can specifically protect PD pathogenesis caused by dysregulation of intracellular calcium homeostasis, calling for future clinical studies of pioglitazone and its analogs to be conducted specifically on PD patients that harbor PINK1 or Parkin mutations.

## Methods

### Plasmid constructs and chemical reagents

Wild-type CISD1 (NM_018464.5), wild-type CISD2 (NM_001008388.5), CISD1 D1 mutant (1–71 amino acid), CISD1 CDGSH deletion mutant (ΔCDGSH), and *Drosophila* CISD (NM_143427.4) were cloned into a pcDNA3.1 zeo (+) C-terminal Myc-tagged vector. CISD1 mutants (K55/68 R double mutant, C72A mutant, and C74A mutant) were generated using a site-directed point mutagenesis method. The N-terminal GST-tagged human Parkin WT and C431S mutant were cloned into a pEBG vector. The C-terminal Myc-tagged human PINK1 WT and 3KD (K219A/D362A/D384A) mutant were cloned into a pcDNA3.1 zeo (+) vector. The N-terminal HA-tagged human ubiquitin was cloned into a pRK5 vector. We obtained bovine IP_3_R1 cloned into a pGFP-C1 vector from Dr. Sang Ki Park (Postech, Korea)^95^. Control siRNA and mouse CISD1 siRNA (ACAACGUAGGACCUCUGAUTT) were purchased from Bioneer (Korea). Cells were treated with antimycin A (Sigma), oligomycin (Sigma), carbobenzoxy-Leu-Leu-leucinal (MG132, Calbiochem), or cycloheximide (CHX, Sigma). Pioglitazone was donated by Dr. Minho Shong and Hyon-Seung Yi (Chungnam National University Hospital, Korea), and used to treat mammalian cells and to feed *Drosophila*.

### Cell culture and transfection

PINK1 WT and KO MEF, Parkin WT and KO MEF, CISD1 WT and KO MEF, HeLa, HEK293, and HEK293T cells were used. All cell lines were cultured in DMEM (Welgene, Korea) supplemented with 10% fetal bovine serum (Invitrogen) at 37 °C in a humidified atmosphere composed of 5% CO_2_. HEK293T cells were transfected using a polyethyleneimine reagent (Sigma). HeLa cells were transfected using Lipofectamine LTX (invitrogen). HEK293 and MEF cells were transfected using Lipofectamine 3000 as instructed by the manufacturer (Invitrogen). For siRNA transfection, we used Lipofectamine RNAiMAX (Invitrogen). We used Lipofectamine 2000 (Invitrogen) for co-transfection of both siRNA and plasmid DNA.

### Generation of CISD1 KO cells

The CRISPR genome editing technique was used for the generation of CISD1 mutant MEF cells. To generate CISD1 KO MEF cells, the guide RNA sequence (GCACAGCGGAGTTGGAGCTG) was cloned into the PX459 vector (Addgene, #62988). We generated CISD1 KO cells using the previously reported method^96^. The plasmid was transfected into MEF cells. 48 hr after transfection, transfected cells were selected by 10 μg/ml puromycin for 3 days, and then single colonies were transferred onto 96-well plates with one colony in each well. The CISD1 KO clones were screened by immunoblot analysis with rabbit anti-CISD1 antibody (Proteintech).

### Antibodies and compounds

Listed with respective commercial information in Supplementary Table 2.

### Immunoprecipitation and immunoblotting

For immunoprecipitation, cells were lysed using Lysis Buffer A (20 mM Tris pH 7.5, 100 mM NaCl, 1 mM EDTA, 2 mM EGTA, 50 mM β-glycerophosphate, 50 mM NaF, 1 mM sodium vanadate, 2 mM DTT, 1 mM PMSF, 10 µg/ml leupeptin, 1 μg/ml pepstatin A, and 1% Triton X-100) and were subjected to immunoprecipitation and immunoblotting according to standard procedures. For immunoblotting for *Drosophila* samples, we homogenized ten 3 day-old male flies using Lysis Buffer B (50 mM Tris pH 8.0, 150 mM NaCl, 1% NP40, 0.5% sodium deoxycholate, and 0.1% SDS). To detect cytosolic calcium signaling in cells, cells were lysed using Lysis Buffer B. Cell or *Drosophila* lysates were centrifuged 15,871 × *g* at 4 °C for 20 min. Samples lysed with Lysis Buffer B were measured for protein concentration using BCA assay kit (Thermo Scientific). Lysates were incubated overnight after the addition of primary antibodies and then incubated with protein A/G agarose beads for 2 hr at 4 °C for immunoprecipitation. The immunoprecipitates were washed 4 times in detergent-free Lysis Buffer A and eluted with 2× Laemmli Buffer at 95 °C. The samples were subjected to SDS-PAGE analysis followed by immunoblotting according to standard procedures. The blots were developed and viewed under LAS-4000 (Fujifilm, Japan). Image J software was used to quantify the protein band intensity.

### Immunofluorescence analysis in mammalian cells

HeLa cells were sub-cultured on coverslips in 12-well tissue culture plates. Cells were transfected with C-terminal Myc-tagged *Drosophila* CISD (CISD), human CISD1 (hCISD1), and human CISD2 (hCISD2) for 24 h, and fixed in 4% paraformaldehyde (Biosesang, Korea) for 15 min. Cells were permeabilized with ice-cold 100% methanol for 20 min and then the cells were incubated in blocking solution (4% BSA and 1% normal goat serum in 0.1% Triton X-100 in 1× PBS) for 1 h at room temperature. Primary antibodies were added to blocking solution and the cells were incubated overnight at 4 °C. After washing with PBST (0.1% Triton X-100 in 1× PBS) for four times, cells were incubated in the blocking solution with secondary antibodies for 1 h at room temperature. The antibody-labeled cells were then washed with PBST for six times and were mounted with mounting solution [100 mg/ml 1,4-diazabicyclo[2.2.2] octane (DABCO) in 90% glycerol]. The prepared slides were observed under LSM710 laser scanning confocal microscope (Carl Zeiss).

### Measurement of calcium levels in mammalian cells

Parkin, PINK1 and CISD1 WT or KO MEF cells were cultured on 10 mm L-poly-lysine coated coverslips embedded in a 24-well plate, and transfected with ER calcium indicator G-CEPIA1er (472 ± 15 nm excitation/520 ± 17.5 nm emission; Addgene, #105012) or cytosol calcium indicator RCaMP1h (543 ± 20 nm excitation/580 ± 20 nm emission; Addgene, #105014) using Lipofectamine 3000. After 48 h of transfection, we monitored the live cells expressing G-CEPIA1er or RCaMP1h using LSM710 laser scanning confocal microscopy (Carl Zeiss, Germany). The cells were placed in a 37 °C heated chamber and perfused with KRB Buffer (140 mM NaCl, 3.6 mM KCl, 0.5 mM NaH_2_PO_4_, 0.5 mM MgSO_4_, 1.5 mM CaCl_2_, 10 mM HEPES, 2 mM NaHCO_3_, 5.5 mM glucose, and pH 7.4-titrated with NaOH). After 2 ~ 3 min of baseline recording, a single pulse of 100 µM ATP was delivered to liberate calcium stores for 3 min and then washed out. In our calcium imaging for pioglitazone treatment, we measured ER calcium release and cytosolic calcium levels after 15 min of pre-treatment with pioglitazone followed by ATP treatment. Peak amplitudes of Ca^2+^ responses to 100 µM ATP were normalized to the basal fluorescence (F_0_) before stimulation. The area-under-the-curve (AUC) of the bar graph was calculated by multiplying the changes in fluorescence over the basal (ΔF/F_0_) by the time (S). Calcium transients were continuously recorded and analyzed on Zen software (Carl Zeiss, Germany).

### Measurement of the influx and efflux of ER calcium in mammalian cells

For this assay, 20 µM β-escin in Intracellular Medium (ICM; 10 mM HEPES, 125 mM KCl, 19 mM NaCl, 1 mM EGTA, and pH 7.3-titrated with KOH) was used for 100 sec to permeabilize cells. After washing with ICM for 5 min, permeabilized cells were superfused for 3 ~ 4 min with Loading Buffer (10 mM HEPES, 125 mM KCl, 19 mM NaCl, 1 mM EGTA, 0.65 mM CaCl_2_, 1.4 mM MgCl_2_, 3 mM Na_2_ATP, and pH 7.3-titrated with KOH) to stimulate SERCA and load Ca^2+^ stores. After that, release buffer (10 mM HEPES, 125 mM KCl, 19 mM NaCl, 1 mM EGTA, 0.65 mM CaCl_2,_ and pH 7.3-titrated with KOH) with 1 µM IP_3_ (850115 P, Sigma) was superfused for 3 ~ 4 min to stimulate IP_3_R. Peak amplitudes of Ca^2+^ responses to solution changes were normalized to the basal fluorescence (F_0_) before stimulations. The ER Ca^2+^ release rate of the bar graph was calculated by dividing the changes in fluorescence over the maximum (ΔF/F_max_) by the time (S).

### Measurement of basal cytosolic calcium levels and ROS levels in mammalian cells

PINK1, Parkin, and CISD1 WT or KO MEF cells were seeded in 6 well plate. For siRNA transfection, we used Lipofectamine RNAiMAX (Invitrogen). After 48 h, for measurement of basal cytosolic calcium level, cells were stained with 10 µM Fluo-3/AM (Invitrogen) in cell media for 30 min at 37 °C in a humidified atmosphere composed of 5% CO_2_. For ROS staining, cells were stained with 10 µM CM-H_2_DCF (Invitrogen) in Hanks’ Balanced Salt Solution (HBSS, Welgene) for 30 min at 37 °C in a humidified atmosphere composed of 5% CO_2_. Cells were harvested with 0.25% trypsin-EDTA solution and were centrifuged at 94 × *g* at 4 °C for 3 min. Cell pellets were resuspended with cold 1× PBS with 2% FBS and measured intensity of Fluo-3/AM and CM-H_2_DCF using FACS analysis. Fluo-3/AM and CM-H_2_DCF was excited with a 488-nm laser. Stained cells were analyzed with a FACS canto II instrument (BD Biosciences) and conducted three independent experiments. BD FACSDiva v.6.1.3 software was used for data analysis.

### Fly stocks

*Drosophila* lines used in the experiments were *hs*-GAL4 (2077; the Bloomington Drosophila Stock Center), *mef2*-GAL4 (27390; the Bloomington Drosophila Stock Center), *PINK1*^*B9*^ (34749; the Bloomington Drosophila Stock Center), *park*^1^ (34747; the Bloomington Drosophila Stock Center), UAS-CISD RNAi (33925 and 104501; the Vienna Drosophila Resource Center), UAS-Itpr (30742; the Bloomington Drosophila Stock Center), UAS-Itpr RNAi (6484; the Vienna Drosophila Resource Center), UAS-ERGCaMP6-210 (83294; the Bloomington Drosophila Stock Center), UAS-GCaMP5G (42037; the Bloomington Drosophila Stock Center), UAS-Luciferase RNAi (31603; the Bloomington Drosophila Stock Center), and UAS-DsRed (6282; the Bloomington Drosophila Stock Center). The UAS-CISD WT-HA was generated by microinjecting pUAST-CISD-HA into *w*^*1118*^ embryos. The RNAi lines for Parkin substrate genes used in Fig. 2a were described in Supplementary Table 1. All *Drosophila* stocks were maintained at 25 °C on the standard cornmeal-yeast-agar medium.

### Generation of CISD KO flies

To generate CISD KO flies, we injected two different plasmids into fly embryos: sgRNA expression vector, and Cas9 expression vector (pHsp700-Cas9; #45945, Addgene). To construct the sgRNA expression vector, 20-bp sgRNA sequence (GGCAGACTTGACAAGTAATT) were synthesized and inserted into pU6-Bbs1-chiRNA vector (#45946, Addgene). After injection, CISD KO flies were identified by PCR and subsequent DNA sequencing.

### Measurement of calcium levels in *Drosophila*

ERGCaMP6-210 was expressed in the muscle by *mef2*-GAL4 and UAS-ERGCaMP6-210 for ER calcium measurement in *Drosophila* larval muscle. GCaMP5G was also expressed by *mef2*-GAL4 and UAS-GCaMP5G for cytosolic calcium measurement. Larvae were dissected in a perfusion buffer (2 mM CaCl_2_, 4 mM MgCl_2_, 2 mM KCl, 2 mM NaCl, 5 mM HEPES, 35.5 mM sucrose, 7 mM l-glutamic acid, and pH 7.3-titrated with NaOH) on a stereomicroscope. After dissection of larval muscle, the larvae were transferred to a confocal microscope and perfused with the same buffer. The fluorescence intensity before stimulation was taken as basal fluorescence (F_0_). To measure the basal calcium levels, the values of F_0_ were normalized to the genetic control. Thapsigargin (T9033, Sigma) and 2-aminoethyl diphenylborinate (D9754, Sigma) were applied for 1 h at each concentration and the fluorescence intensity was measured. Next, in order to measure the stimulation changes by ATP, F_0_ was recorded for 2 ~ 3 min. Then, a single pulse of 5 mM ATP was delivered to liberate calcium stores for 3 min and washed out. Peak amplitudes of Ca^2+^ responses to 5 mM ATP were normalized to the basal fluorescence (F_0_) before stimulation. The area-under-the-curve (AUC) of the bar graph was calculated by multiplying the changes in fluorescence over the basal (ΔF/F_0_) by the time (S). In our calcium assays for pioglitazone treatment, we measured ER calcium release and cytosolic calcium levels after 15 min of pre-treatment with 1 mM pioglitazone followed by ATP treatment. Fluorescence images were acquired by using an IX-73 inverted microscope platform (Olympus, Japan) with a camera (Prime-BSI CMOS camera, Teledyne Photometrics) attachment and an illuminator (pe-340Fura, CoolLED, UK). The ratio fluorescence responses of ERGCaMP6-210 (480 ± 10 nm excitation/510 ± 10 nm emission) or GCaMP5G (480 ± 10 nm excitation/510 ± 10 nm emission) were analyzed using Metafluor 6.3 software (Molecular Devices).

### Measurement of the influx and efflux of ER calcium in *Drosophila*

After obtaining the larval muscle of flies expressing ERGCaMP6-210 in the same manner as described above, 200 µM β-escin in ICM was treated for 10 min for permeabilization. After washing with ICM for 5 min, permeabilized tissues were superfused for 3 ~ 4 min with Loading Buffer (5 mM HEPES, 2 mM KCl, 2 mM NaCl, 1 mM EGTA, 2 mM CaCl_2_, 4 mM MgCl_2_, 3 mM Na_2_ATP, and pH 7.3-titrated with KOH) to stimulate SERCA and load Ca^2+^ stores. After that, release buffer (5 mM HEPES, 2 mM KCl, 2 mM NaCl, 1 mM EGTA, 2 mM CaCl_2,_ and pH 7.3-titrated with KOH) with 5 µM IP_3_ was superfused for 3 ~ 4 min to stimulate IP_3_R. Peak amplitudes of Ca^2+^ responses to solution changes were normalized to the basal fluorescence (F_0_) before stimulations. The ER Ca^2+^ release rate of the bar graph was calculated by dividing the changes in fluorescence over the maximum (ΔF/F_max_) by the time (S).

### Measurement of ROS levels in *Drosophila*

Five 3-day-old male flies were dissected in 1× PBS buffer and their thoraces were collected. After incubation in 30 µM dihydroethidium (DHE, D1168, Invitrogen) for 7 min in a dark chamber, samples were washed 3 times in 1× PBS buffer. Next, samples were fixed for 8 min in 4% PFA and washed three times for 15 min each in 1× PBS buffer. Then, samples were stained with Hoechst (1:200, Hoechst 33258, Invitrogen) for 15 min at room temperature to detect the nucleus. Samples were mounted with SlowFade mounting solution (S36936, Invitrogen) after 3 washes in 1× PBS buffer. The prepared slides were observed under LSM710 laser scanning confocal microscope (Carl Zeiss, Germany). Quantification of fluorescence intensity was performed using ImageJ software.

### Quantification of *Drosophila* thorax and wing abnormality

For quantification of the abnormality of thorax and wing, the percentage of 3-day-old male flies showing defective thoraces and upturned or downturned wings were measured out of ten flies. To quantify, ten independent experiments were performed. Finally, the percentage of normal phenotype was calculated out of 100 flies.

### Immunohistochemistry of *Drosophila* thorax

To observe the adult fly thorax, the fly head was removed from 3-day-old male flies and the remaining body was fixed with 4% paraformaldehyde (PFA) for 1 h and washed out 3 times with PBST for 10 min, then it was dissected to get the thorax. The fly thoraces were permeabilized with 0.5% Triton X-100 in 1× PBS for 5 min and washed out 3 times with PBST. After that, the thoraces were incubated with 3% bovine serum albumin in PBST for 30 min at room temperature. Streptavidin (1:200, Alexa Fluor 488 streptavidin, Invitrogen) and phalloidin (1:200, phalloidin-tetramethylrhodamine B isothiocyanate, Merck) were applied for overnight at 4 °C to stain mitochondria and thorax muscle actin filament, respectively. On the next day, the thoraces were washed out 3 times with PBST and mounted on a slide glass with SlowFade mounting solution (S36936, Invitrogen). To quantify the percentage of flies having mitochondrial abnormalities, we defined the flies having the mitochondria over length 5 µM and width 3 µM as the flies having abnormal mitochondria. Ten 3-day-old male flies were counted for the percentages, and ten independent experiments were performed for quantification.

### TUNEL assay in *Drosophila*

Collected thoraces according to the method described above were incubated with 0.1 M sodium citrate in PBST for 30 min at 65 °C, and cell death was detected using in situ cell death detection kit (Roche Applied Science). After the TUNEL reaction, the thoraces were stained by Hoechst (1:200, Hoechst 33258, Invitrogen) to detect the nucleus for 15 min at room temperature. To quantify the percentage of flies showing apoptosis, we defined the flies having more than ten TUNEL dots as the fly with apoptosis^31^. For the percentages, ten 3-day-old male flies were counted, and ten independent experiments were performed for the data quantification.

### Climbing assay in *Drosophila*

To conduct the climbing assay, ten 3-day-old male flies were transferred into 18-cm-long vials and incubated for 30 min at room temperature for the acclimatization period. After all flies were moved completely down to the bottom by gently tapping, we measured the time of their climbing at the 15-cm finish line when more than five flies had arrived. Three trials were performed for each group, and ten independent experiments were performed. The average climbing time was calculated for each genotype.

### DA neuron staining in *Drosophila*

30-day-old male flies were fixed with 4% paraformaldehyde (PFA) for 3 h and washed out 3 times with PBST for 10 min. Next, the brains were dissected and permeabilized with 0.5% Triton X-100 PBST for 5 min, and washed out 3 times with PBST. After 30 min of incubation with 3% bovine serum albumin in PBST at room temperature, DA neurons were stained with anti-tyrosine hydroxylase (TH) mouse antibody (1:200, Immunostar) at 4 °C for 48 h. DA neurons were observed by LSM710 laser scanning confocal microscopy (Carl Zeiss, Germany) via Z-stack analysis. We counted the number of DA neurons of ten flies from each genotype.

### Quantitative real-time PCR in *Drosophila*

Total RNA was extracted from ten 3-day-old male fly bodies with TRIzol (Invitrogen), and RNA samples were reverse-transcribed by M-MLV reverse transcriptase (Primega). Relative quantification PCR was carried out using a SYBR TOPreal^TM^ qPCR 2× PreMIX (Enzynomics), and a CFX96^TM^ Real-Time System (BIO-RAD). Rp49 and Actin 5 C were used as internal controls, and gene expression levels were normalized to the controls. Following primers were used: Rp49 (F 5′-AGCTTCAAGATGACCATCCG-3′, and R 5′-CCAGGAACTTCTTGAATCCG-3′), Actin 5 C (F 5′-TGTGACGAAGAAGTTGCTGC-3′, and R 5′-TCATCACCCACGTACGAGTC-3′), and IP_3_R (F 5′-GCGGCTTGGGATTACTAGGCCTG-3′, and R 5′-CATCATCCGACCCGGCGTCC-3′). For the data quantification, measurements were conducted in triplicates.

### Statistical analysis

A blind manner was used in all experiments and analyses. Image areas were randomly selected during observing samples. For computing *p* values, one-way ANOVA (Tukey’s multiple comparison test), one-sided Chi-square test, and two-tailed unpaired Student’s *t*-test were used. All tests were examined via GraphPad Prism v.9 (GraphPad Software) for the statistics.

### Reporting summary

Further information on research design is available in the Nature Portfolio Reporting Summary linked to this article.

### Supplementary information

Supplementary Information

Reporting Summary

### Source data

Source Data

## Data Availability

A Reporting Summary for this article is available as Supplementary Information file. The main data supporting the findings of this study are available within the article and its Supplementary Figures. The source data for Figs. 1–8 and Supplementary Figs 1–16 are provided as a Source Data file. Specific data *p* values are also included within the Source Data file. Additional details on datasets and protocols that support the findings of this study will be made available by the corresponding author upon request. Source data are provided with this paper.
