# Supplementary material for: PINK1 and Parkin regulate IP3R-mediated ER calcium release
Source: Nat Commun. 2023 Aug 25;14:5202. doi: 10.1038/s41467-023-40929-z (PMC10457342; doi:10.1038/s41467-023-40929-z)
Supplement: Supplementary file 1 — Supplementary Information [file 41467_2023_40929_MOESM1_ESM.pdf]

PINK1 and Parkin regulate IP_3R-mediated ER calcium release

Su Jin Ham, Heesuk Yoo, Daihn Woo, Da Hyun Lee, Kyu-Sang Park and Jongkyeong Chung

Supplementary Information

Supplementary information includes 16 Supplementary Figures and 2 Supplementary Table

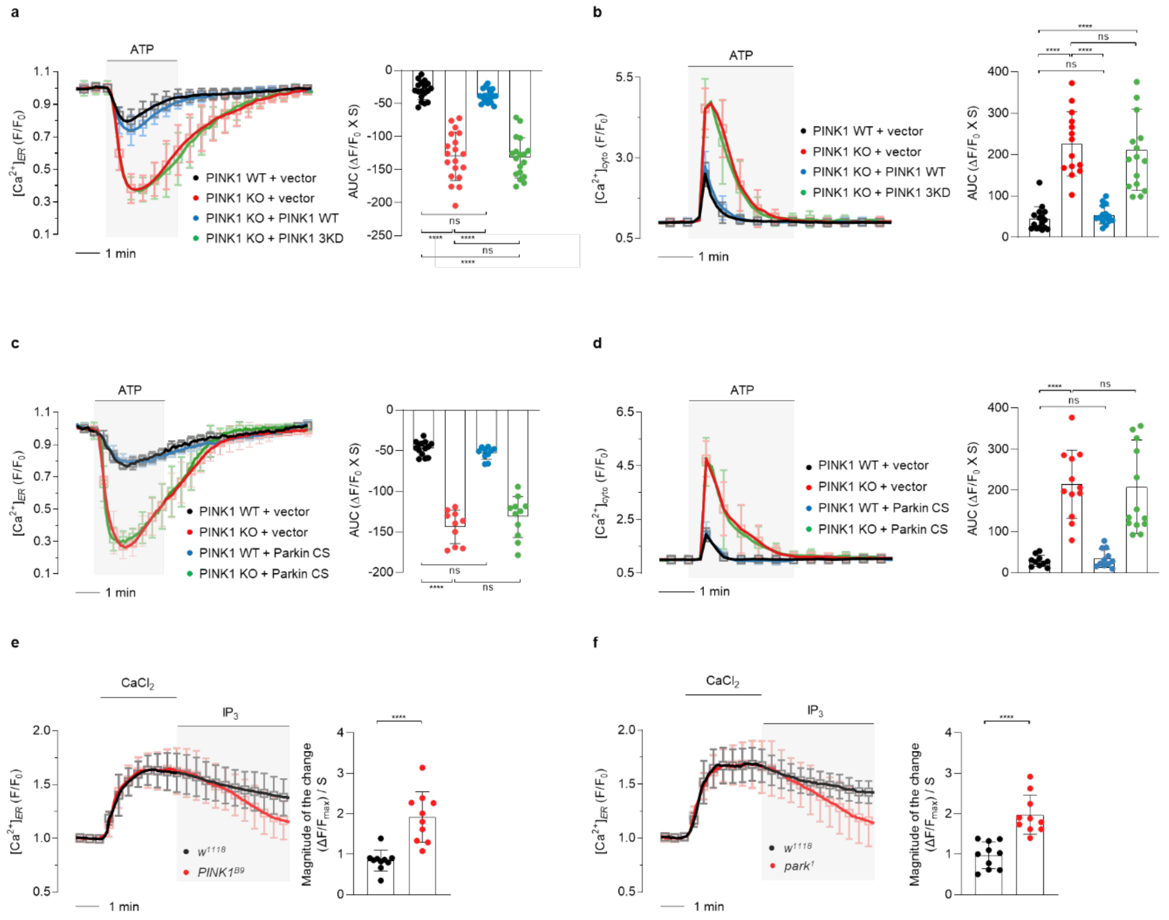

Supplementary Figure 1. The activity of PINK1 and Parkin signaling pathway modulates ER calcium release (Related to Figure 1).

a-b, Measurement of ER (**a**) and cytosolic (**b**) calcium modulation in PINK1 KO MEF cells when expressing PINK1 WT, 3KD (K219A/D362A/D384A) mutant, or control empty vector (vector). 100 μM ATP was delivered to initiate IP_3R-mediated calcium release. n = 108~142 cells. **c-d**, Measurement of ER (**c**) and cytosolic (**d**) calcium modulation in PINK1 MEF cells when expressing Parkin CS (C431S) mutant or empty vector (vector). n = 114~133 cells. **e-f,** Measurement of ER calcium release in w^{1118} and *PINK1*^{B9} (**e**) and *park*^{1} **(f)** flies. The bar graphs indicate the magnitude of the change during IP_3 treatment. n = 10 flies. Three independent experiments were conducted and quantified (**a-f**). One-way analysis of variance (ANOVA) with Tukey's multiple comparisons test was used (**a-d**) and two-tailed unpaired Student's *t*-test was used (**e** and **f**). **** represents $p < 0.0001$ and ns represents not

significant. Source data, the exact *p* values, and *n* number of each experiment are included within the Source Data file. All data are presented as mean ± SD.

a

Gene	Flybase	Human homolog gene	RNAi#	Lethality
CISD	CG1458	CISD1, 2	v33925	-
porin	CG6647	VDAC1, 2, 3	v330138	-
Cth	CG5345	CTH	v103779	-
eIF2B-beta	CG2677	EIF2B2	v25426	-
AlaRS	CG13391	AARS1	v39776	O
Sply	CG8946	SGPL1	v103485	O
rdhB	CG7077	DHRS11	v102434	O
AdSS	CG17273	ADSS, ADSSL1	v29520	O
Tom70	CG6756	TOMM70	v18112	-
Pgls	CG17333	PGLS	v100734	-
Vps4	CG6842	VPS4A, B	v105977	O
Syx5	CG4214	STX5	v108928	O
Mpcp2	CG4994	SLC25A3	v101316	O
Vps35	CG5625	VPS35	v22180	-
eIF2-alpha	CG9946	EIF2S1	v7798	-
CG11396	CG11396	TRAPPC12	v103546	O
sesB	CG16944	SLC25A4, 5, 6, 31	v104576	-
CG13887	CG13887	BCAP31, 29	v106452	O
Rdh1	CG2064	RDH11	v103276	-
CG8839	CG8839	FAAH2	v104479	-
Vha26	CG1088	ATP6V1E1, 2	v102378	O
Cyp4p1	CG10842	CYP4V2, A22, B1, F11, F22, CYP20A1	v3331	-
Idh	CG7176	IDH1, 2	v100554	-
ALiX	CG12876	PDCD6IP	v32047	-
Pp2B-14D	CG9842	PPP3CA, B, C	v46873	O
CG4199	CG4199	AIFM3	v26424	-
Cat	CG6871	CAT	v103591	O
Pdh	CG4899	HPGD	v104428	O
Vha55	CG17369	ATP6V1B2, 1	v46554	-
CCT4	CG5525	CCT4	v106099	O
Vha44	CG8048	ATP6V1C1, 2	v101527	-
Ifrd1	CG31694	IFRD1, 2	v21398	-

b

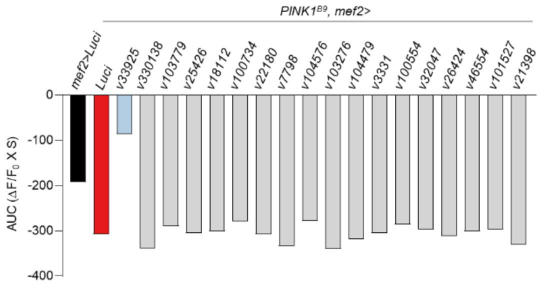

c

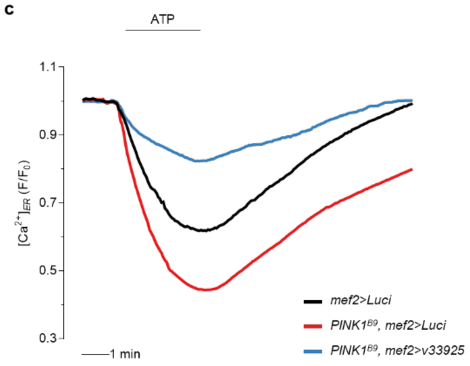

Supplementary Figure 2. Results of the genetic screening for the regulators of ER calcium release in Fig. 2a (Related to Figure 2).

a, List of the genes screened for the regulators of ER calcium mobilization. When these candidate RNAi were expressed by *mef2*-GAL4 that induces gene expression in the muscle of *Drosophila*, 14 RNAi showed lethal phenotypes. We therefore conducted calcium imaging experiments with the remaining 18 candidate gene RNAi lines. **b-c**, Measurement of ER calcium release in the PINK1 null background flies (*PINK1*^{B9}) with *mef2*-GAL4-induced expression of each candidate RNAi or control Luciferase RNAi (*Luci*). 5 mM ATP was delivered to initiate ER calcium release in *Drosophila* larval muscle. Source data is included within the Source Data file.

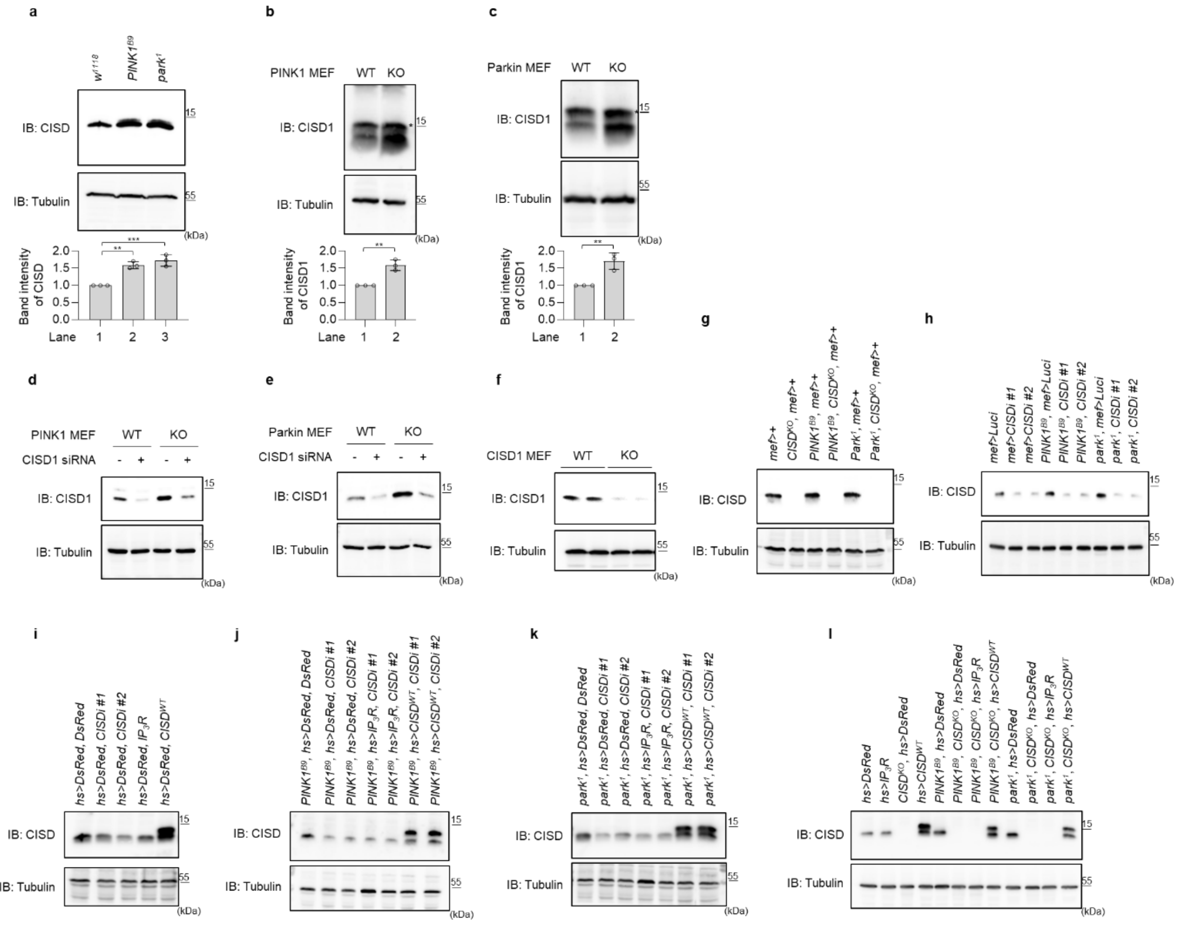

Supplementary Figure 3. Endogenous CISD1/CISD protein levels in MEF cells and *Drosophila* (Related to Figure 2, 3, 4, 5, and Supplementary Figure 4, 5, 7, 8, 10, 11, 15).

a, Immunoblot analysis of endogenous CISD in w^{1118}, PINK1 null (*$PINK1^{B9}$*), and Parkin null (*$park^{1}$*) flies. **b-c**, Immunoblot analysis of endogenous CISD1 in WT and PINK1 KO MEF cells (**b**) and WT and Parkin KO MEF cells (**c**). Three independent experiments were conducted and the immunoblot band intensity for CISD1 were quantitated (bar graphs in bottom, **a-c**). Data are presented as mean ± SD. **d-e**, Immunoblot analysis of endogenous CISD1 in WT and PINK1 KO MEF cells (**d**) and WT and Parkin KO MEF cells (**e**) transfected with control siRNA or CISD1 siRNA. **f**, Immunoblot analysis of endogenous CISD1 in WT and CISD1 KO MEF cells. **g-l**, Immunoblot analysis of endogenous CISD in the flies with indicated genotypes. Three independent experiments were conducted and were quantified (**d-f**). Two biological replicates were performed for each experimental condition (**g-l**). Quantification graph are included within the Source Data file (**d-l**). One-way analysis of variance

(ANOVA) with Tukey's multiple comparisons test was used (**a**) and two-tailed unpaired Student's *t*-test was used (**b** and **c**). *** represents $p < 0.001$ and ** represents $p < 0.01$. Source data and the exact *p* values are included within the Source Data file (**a-l**).

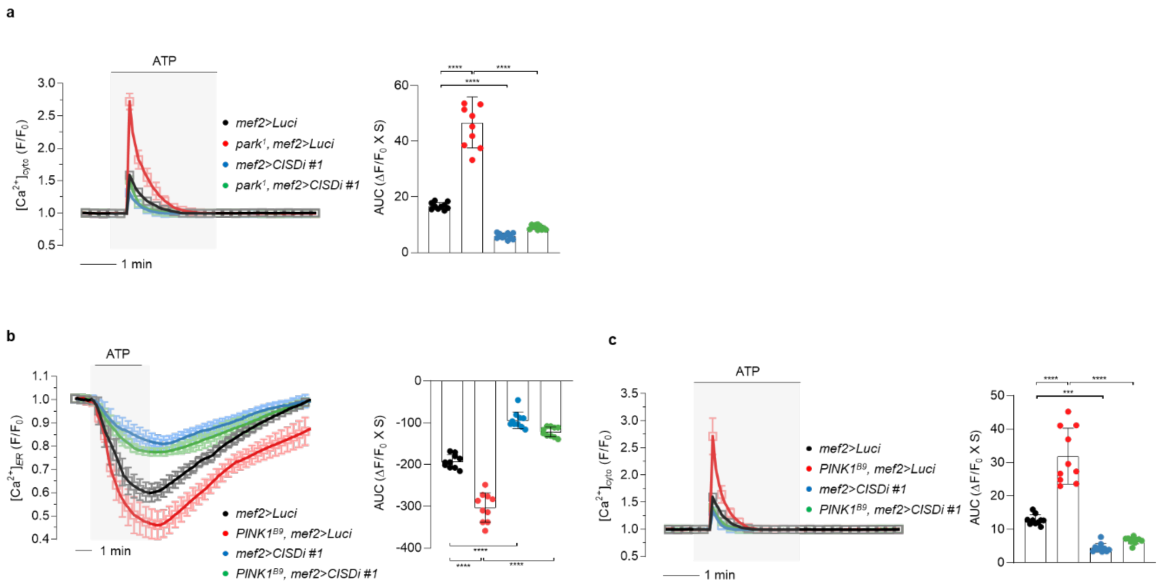

Supplementary Figure 4. CISD knockdown rescues the increased cytosolic calcium levels and ER calcium release in PINK1- or Parkin-deficient flies (Related to Figure 2).

a, Measurement of cytosolic calcium modulations in control (*mef2>Luci*, black) and Parkin null mutant flies (*park^{1}*, red). Similar experiments were also conducted in CISD knockdown flies (*CISDi #1*, blue) and Parkin null flies expressing CISD RNAi (*park^{1}*, *CISDi #1*) (green). We obtained #33925 *CISDi #1* RNAi line from the Vienna Drosophila Resource Center. The right side bar graphs indicate the quantification of the normalized calcium traces using AUC of calcium release during ATP treatment. n = 10 flies. **b-c**, Measurement of ER (**b**) and cytosolic (**c**) calcium modulation in control (*mef2> Luci*, black) and PINK1 null mutants (*PINK1^{B9}*, red). Similar experiments were also conducted in CISD knockdown flies (*CISDi #1*, blue) and PINK1 null flies expressing CISD RNAi (*PINK1^{B9}*, *CISDi #1*) (green). The right side bar graphs indicate the quantification of the normalized calcium traces using AUC of calcium release during ATP treatment. n = 10 flies. Three independent experiments were conducted and were quantified (**a-c**). One-way ANOVA with Tukey's multiple comparisons test was used (**a-c**). **** represents $p < 0.0001$ and *** represents $p < 0.001$. Source data and the exact p values are included within the Source Data file. All data are presented as mean ± SD.

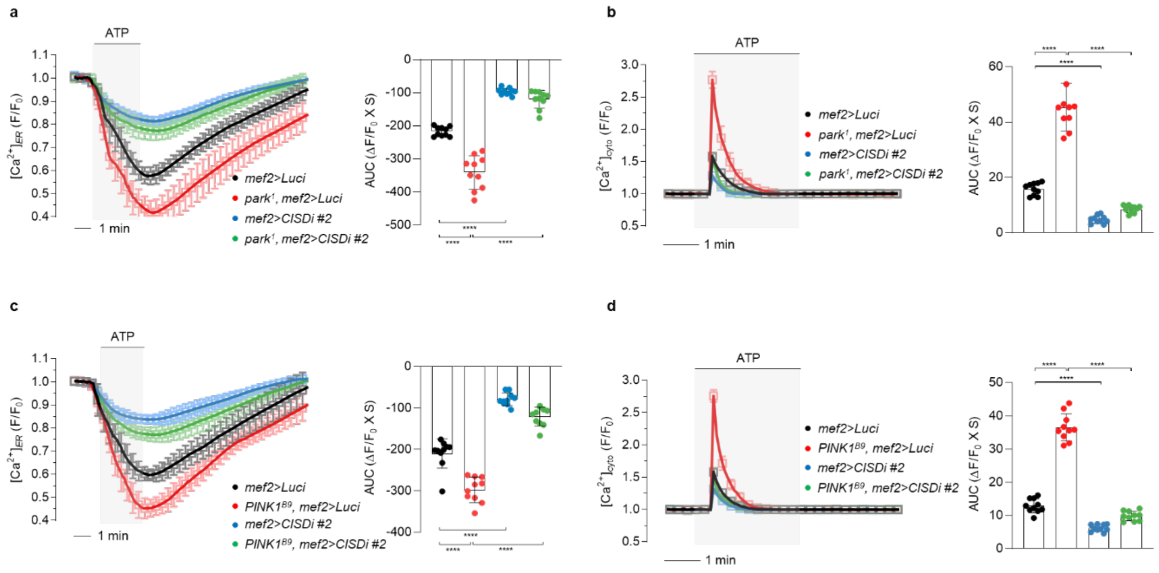

Supplementary Figure 5. CISD knockdown rescues the increased ER calcium release and cytosolic calcium levels in PINK1- or Parkin-deficient flies (Related to Figure 2).

a-b, Measurement of ER (**a**) and cytosolic (**b**) calcium modulation in control (*mef2>Luci*, black) and Parkin null mutants (*park*^{1}, red). Similar experiments were also conducted in CISD knockdown flies (*CISDi #2*, blue) and Parkin null flies expressing CISD RNAi (*park*^{1}, *CISDi #2*) (green). We obtained #104501 *CISDi #2* RNAi flies from the Vienna Drosophila Resource Center. The right side bar graphs indicate the quantification of the normalized calcium traces using AUC of calcium release during ATP treatment. n = 10 flies. **c-d**, Measurement of ER (**c**) and cytosolic (**d**) calcium modulation in control (*mef2>Luci*, black) and PINK1 null mutants (*PINK1*^{B9}, red). Similar experiments were also conducted in CISD knockdown flies (*CISDi #2*, blue) and PINK1 null flies expressing CISD RNAi (*PINK1*^{B9}, *CISDi #2*) (green). The right side bar graphs indicate the quantification of the normalized calcium traces using AUC of calcium release during ATP treatment. n = 10 flies. Three independent experiments were conducted and were quantified (**a-d**). One-way ANOVA with Tukey's multiple comparisons test was used (**a-d**). **** represents $p < 0.0001$. Source data and the exact p values are included within the Source Data file. All data are presented as mean ± SD.

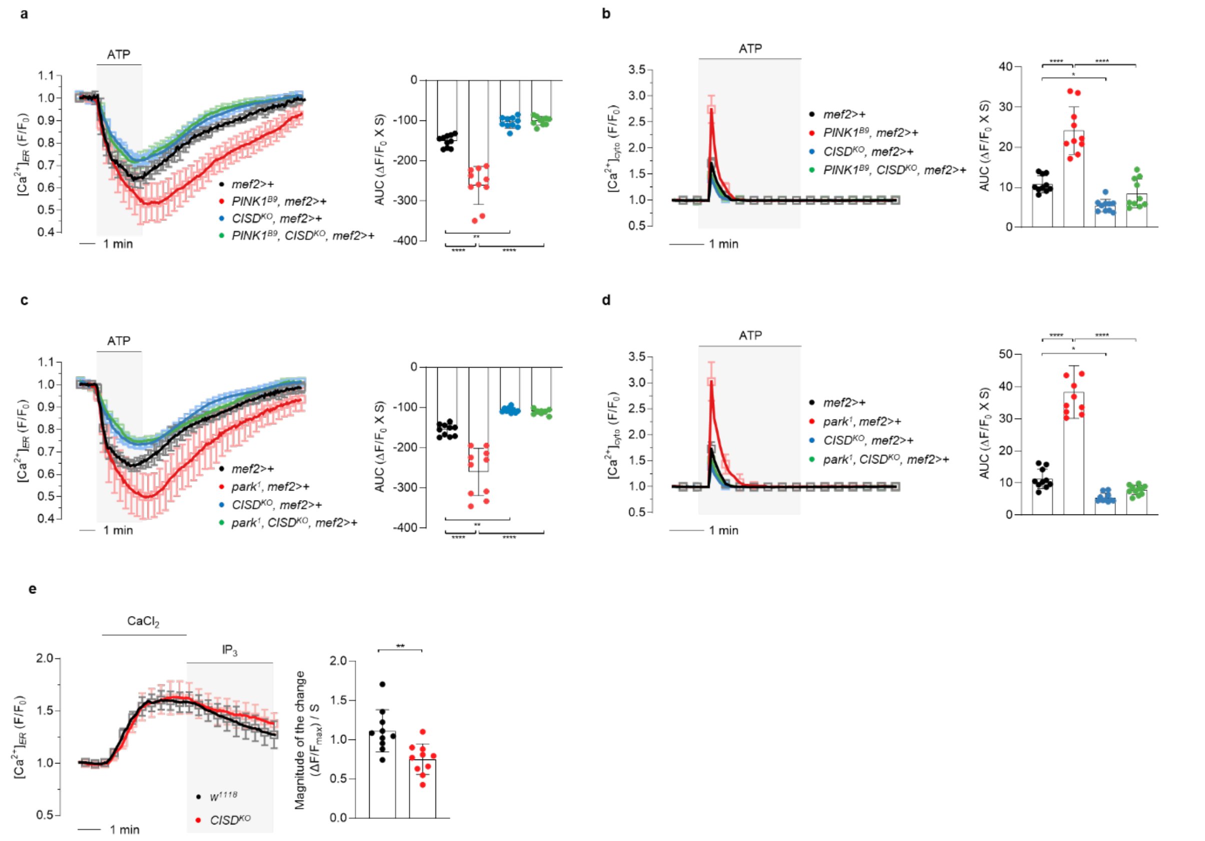

Supplementary Figure 6. Loss of CISD reduced IP$_3$R activity in *Drosophila* (Related to Figure 2).

a-b, Measurement of ER (**a**) and cytosolic (**b**) calcium modulation in control (*mef2*>+, black) and PINK1 null mutants (*PINK1^{B9}*, red). Similar experiments were also conducted in CISD knockout flies (*CISD^{KO}*, blue) and PINK1 null flies crossing with CISD KO flies (*PINK1^{B9}*, *CISD^{KO}*) (green). The right side bar graphs indicate the quantification of the normalized calcium traces using AUC of calcium release during ATP treatment. n = 10 flies. **c-d**, Measurement of ER (**c**) and cytosolic (**d**) calcium modulation in control (*mef2*>+, black) and Parkin null mutants (*park^{1}*, red). Similar experiments were also conducted in CISD knockout flies (*CISD^{KO}*, blue) and Parkin null flies crossing with CISD KO flies (*park^{1}*, *CISD^{KO}*) (green). The right side bar graphs indicate the quantification of the normalized calcium traces using AUC of calcium release during ATP treatment. n = 10 flies (**c**). n = 10 ~ 11 flies (**d**). **e**, Measurement of IP$_3$R activity. The larval muscles of flies were permeabilized for 10 min with 200 μM β-escin in intracellular medium (ICM), and washed with ICM for 5 min. Following that, 0.65

mM $CaCl_2$ was added to estimate the influx of ER calcium. After a steady-state was achieved, 5 µM IP_3 was introduced to evoke ER calcium release to estimate the efflux of ER calcium**.** Measurement of ER calcium release in w^{1118} (black) and $CISD^{KO}$ (red) flies was conducted. The bar graphs indicate the magnitude of the change during IP_3 treatment. $n = 10$ flies. Three independent experiments were conducted and were quantified (**a**-**e**). One-way analysis of variance (ANOVA) with Tukey's multiple comparisons test was used (**a**-**d**), and two-tailed unpaired Student's *t*-test was used (**e**). **** represents $p < 0.0001$, ** represents $p < 0.01$, and * represents $p < 0.05$. Source data, the exact *p* values, and *n* number of each experiment are included within the Source Data file. All data are presented as mean ± SD.

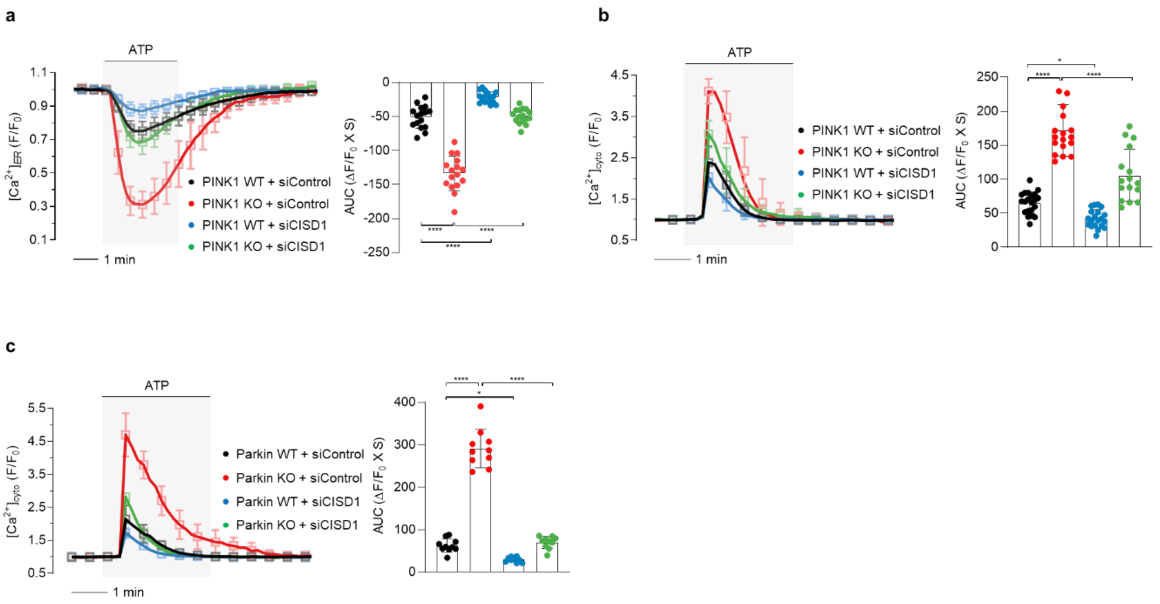

Supplementary Figure 7. CISD1 knockdown rescues the increased ER calcium release and cytosolic calcium levels in PINK1- and Parkin-deficient cells (Related to Figure 2).

a-b, Measurement of ER (**a**) and cytosolic (**b**) calcium modulation in WT (black) and PINK1 KO (red) MEF cells expressing siControl. Similar experiments were also conducted for WT (blue) and PINK1 KO (green) MEF cells expressing siCISD1. The right side bar graphs indicate the quantification of the normalized calcium traces using AUC of calcium release during ATP treatment. *n* = 109~136 cells. **c**, Measurement of cytosolic calcium modulations in WT (black) and Parkin KO (red) MEF cells expressing siControl. Similar experiments were also conducted for WT (blue) and Parkin KO (green) MEF cells expressing siCISD1. The right side bar graphs indicate the quantification of the normalized calcium traces using AUC of calcium release during ATP treatment. *n* = 108~131 cells. Three independent experiments were conducted and were quantified (**a-c**). All statistical significances were determined using a one-way analysis of variance (ANOVA) with Tukey's multiple comparisons test. **** represents $p < 0.0001$ and * represents $p < 0.05$. Source data, the exact *p* values, and *n* number of each experiment are also included within the Source Data file. All data are presented as mean ± SD.

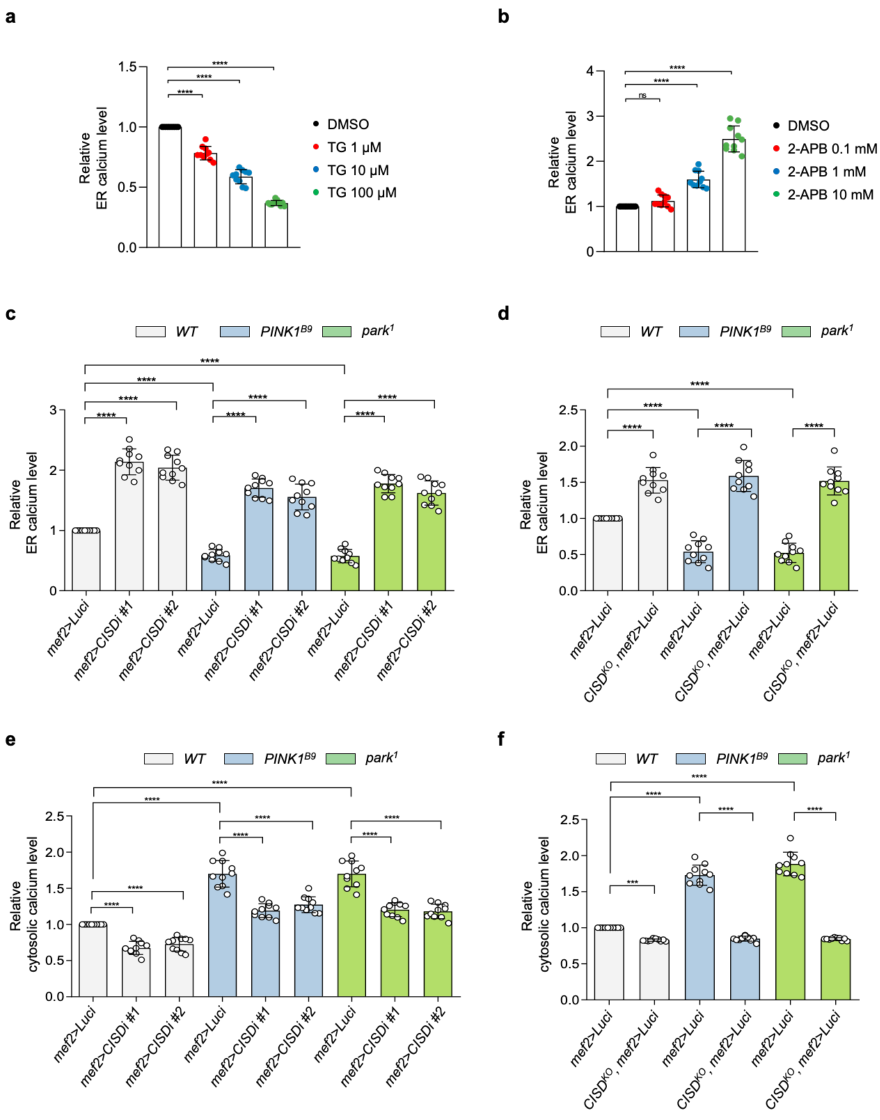

Supplementary Figure 8. CISD controls basal ER and cytosolic calcium levels in *Drosophila* (Related to Figure 3).

a-b, Measurement of basal ER calcium levels when treated with SERCA inhibitor thapsigargin (TG, **a**) and IP_3R inhibitor 2-aminoethyl diphenylborinate (2-APB, **b**) as the indicated concentrations. *n* = 10 flies. **c-d**, Measurement of basal ER calcium levels for control *(mef2>Luci*), PINK1 null mutants (*PINK1^{B9}*), Parkin null mutants (*park^{1}*), CISD knockdown flies (*CISDi #1* and *CISDi #2*), CISD null mutants (*CISD^{KO}*), and PINK1 or Parkin null mutants expressing CISD RNAi (**c**) or crossed with CISD knockout (**d**) flies. *n* = 10 flies. **e-f**, Measurement of basal cytosolic calcium levels for control

(*mef2>Luci*), PINK1 null mutants (*PINK1*^{B9}), Parkin null mutants (*park*^{1}), CISD knockdown flies (*CISDi #1* and *CISDi #2*), CISD null mutants (*CISD*^{KO}), and PINK1 or Parkin null mutants expressing CISD RNAi (**e**) or crossed with CISD knockout (**f**) flies. Bar graphs indicate relative basal calcium levels normalized to basal calcium levels of *mef2*-GAL4 flies. $n = 10$ flies. All statistical significances were determined using a one-way analysis of variance (ANOVA) with Tukey's multiple comparisons test (**a**-**f**). **** represents $p < 0.0001$, *** represents $p < 0.001$, and ns presents not significant. Source data and the exact p values are included within the Source Data file. All data are presented as mean ± SD.

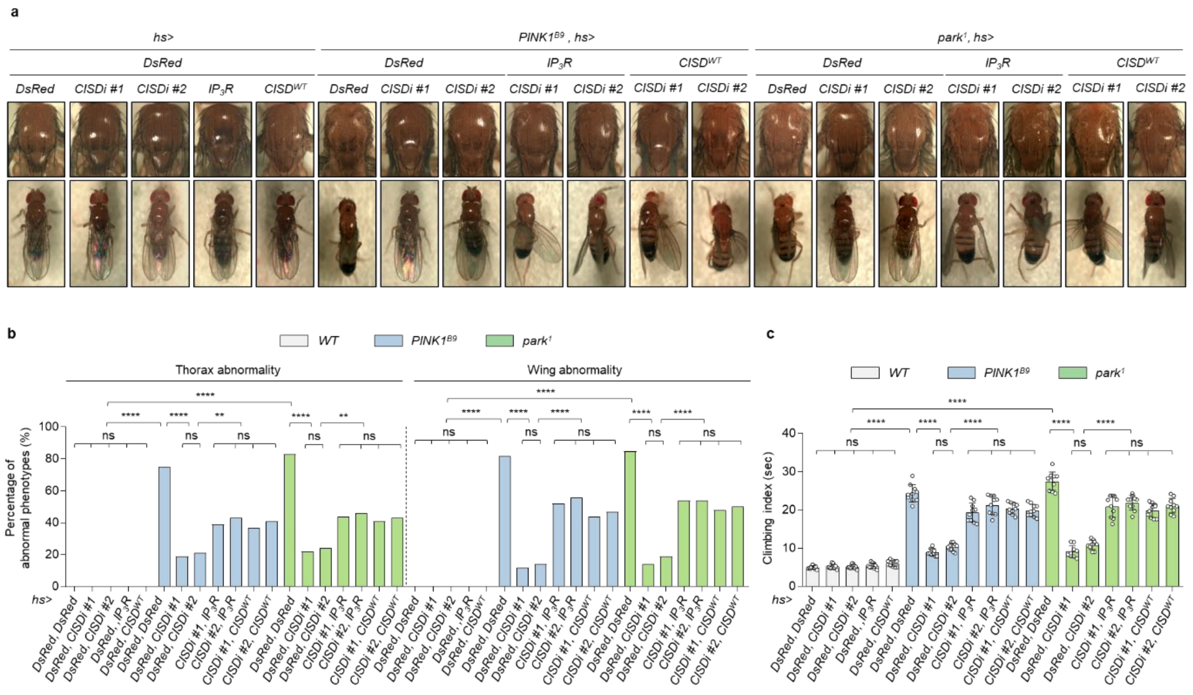

Supplementary Figure 9. Simultaneous expression of WT CISD or IP$_3$R reverse the rescuing effect of CISD knockdown in PINK1 and Parkin null flies (Related to Figure 5).

a, Representative images of *Drosophila* thoracic (top panels) and wing posture (bottom panels) phenotypes. **b**, Quantification of the percentage of flies having abnormal thorax and wing phenotypes. **c**, Measurement of the climbing ability in the adult flies with indicated genotypes. n = 10. One-sided Chi-square test was used (**b**). One-way ANOVA with Tukey's multiple comparisons test was used (**c**). **** represents $p < 0.0001$. ns represents not significant. Source data and the exact p values are included within the Source Data file. All data are presented as mean ± SD.

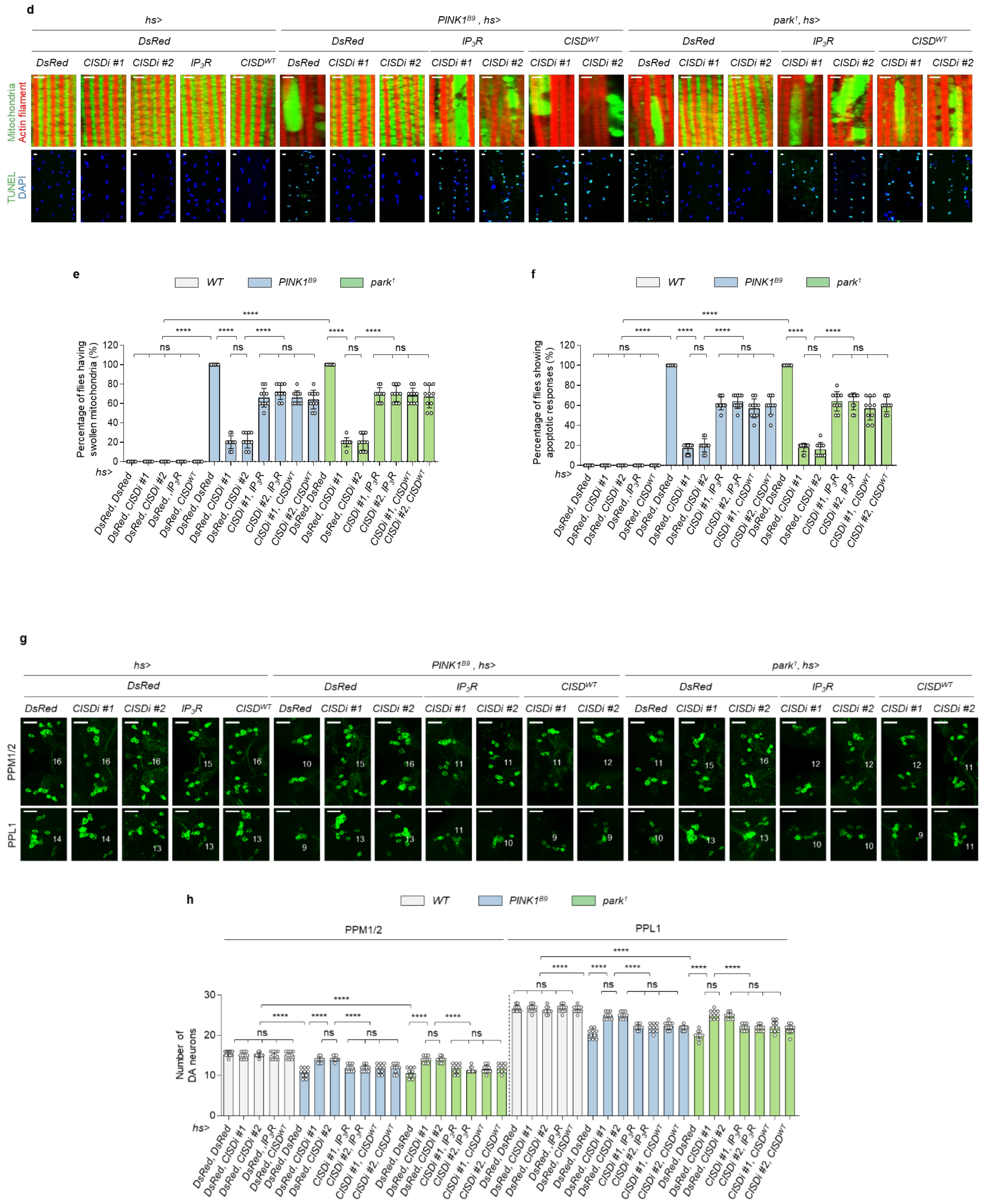

Supplementary Figure 9. Simultaneous expression of WT CISD or IP$_3$R reverse the rescuing effect of CISD knockdown in PINK1 and Parkin null flies (Related to Figure 5).

d, Immunofluorescence images of the adult flight muscles (top). Green (mitochondria) and red (actin filament). Scale bars, 5 μm. TUNEL assays of the adult flight muscles (bottom). Green (TUNEL) and blue (DAPI). Scale bars, 5 μm. **e**, Quantification of the percentage of flies having swollen mitochondria, and the percentage of flies showing apoptotic responses. $n = 10$. **f**, Quantification of the percentage of flies showing apoptotic responses. $n = 10$. **g-h,** Immunofluorescence images (**g**) and number (**h**) of DA neurons in PPM1/2, and PPL1 regions of adult fly brains. Green (DA neurons). Images of PPL1 regions were obtained from one of the left or right side of the PPL1 regions. Numbers of the DA neurons in the PPL1 regions were counted from both hemispheres. $n = 10$. Scale bars, 20 μm. For statistical analysis, Chi-square test was used (**b**). One-way analysis of variance (ANOVA) with Tukey's multiple comparisons test was used (**e**, **f**, and **h**). **** represents $p < 0.0001$, ** represents $p < 0.01$, and ns represents not significant. Source data and the exact p values are included within the Source Data file. All data are presented as mean ± SD.

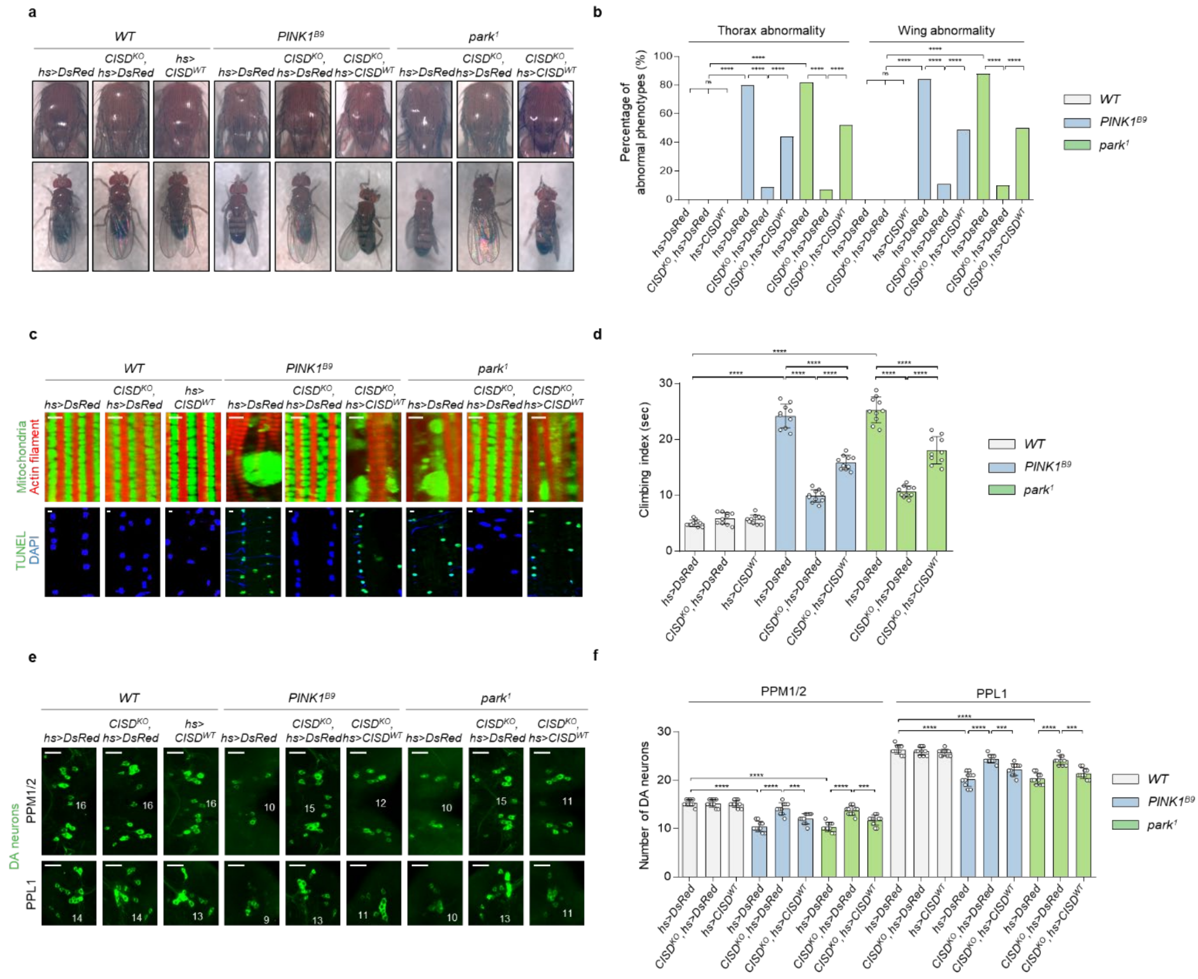

Supplementary Figure 10. Simultaneous expression of WT CISD reverses the rescuing effect of CISD knockout in PINK1 and Parkin null flies (Related to Figure 5).

a-b, Images of *Drosophila* thoracic (top panels) and wing posture (bottom panels) phenotypes (**a**) and percentages of the flies having abnormal phenotypes (**b**). **c**, Immunofluorescence images of the adult flight muscles (top). Green (mitochondria) and red (actin filament). Scale bar, 5 µm. TUNEL assays of the adult flight muscles (bottom). Green (TUNEL) and blue (DAPI). Scale bars, 5 µm. **d**, Measurement of the climbing ability in the adult flies. $n = 10$. **e-f,** Immunofluorescence images (**e**) and number (**f**) of DA neurons in PPM1/2 (top), and PPL1 (bottom) regions of adult fly brains. Green (DA neurons). Images of PPL1 regions were obtained from one of the left or right side of the PPL1 regions. Numbers of the DA neurons in the PPL1 regions were counted from both hemispheres. $n = 10$. Scale bars, 20 µm. Except the genetic results testing for the expression of exogenous WT CISD, the other results were the

same as the results of Fig. 5, because Fig. 5 and Supplementary Fig. 10 were experimented together. For statistical analysis, one-sided Chi-square test was used (**b**). One-way analysis of variance (ANOVA) with Tukey's multiple comparisons test was used (**d** and **f**). **** represents $p < 0.0001$, *** represents $p < 0.001$, and ns represents not significant. Source data and the exact *p* values are included within the Source Data file. All data are presented as mean ± SD.

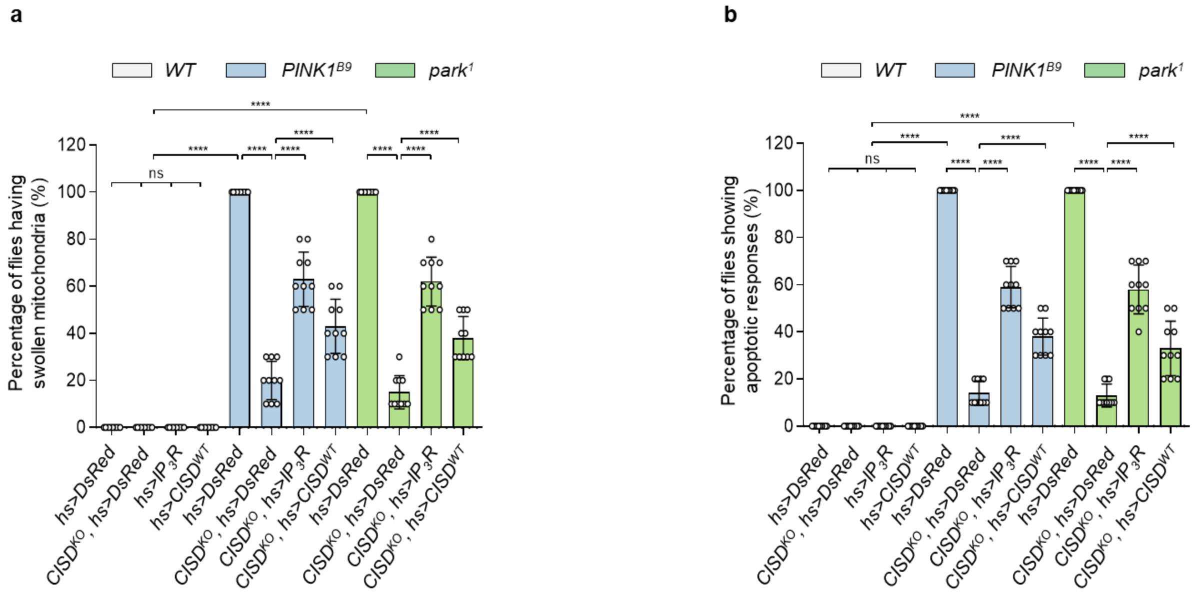

Supplementary Figure 11. Simultaneous expression of IP_3R or WT CISD in PINK1 and Parkin null flies crossed with $CISD^{KO}$ flies reverses the rescuing effect of CISD KO (Related to Figure 5).

a, Quantification of the percentage of flies having swollen mitochondria. $n = 10$. **b**, Quantification of the percentage of flies showing apoptotic responses. $n = 10$. All statistical significances were determined using one-way analysis of variance (ANOVA) with Tukey's multiple comparisons test. **** represents $p < 0.0001$ and ns represents not significant. Source data and the exact p values are included within the Source Data file. All data are presented as mean ± SD.

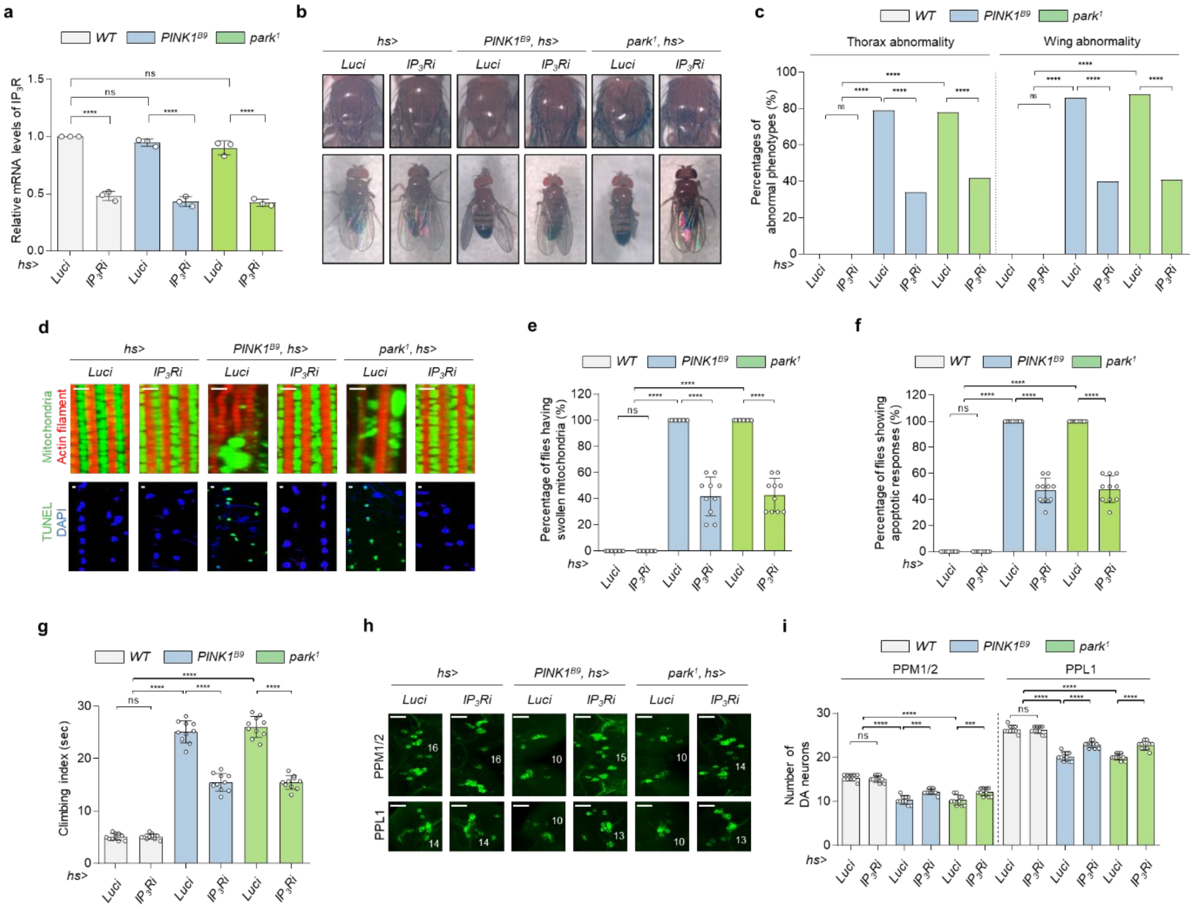

Supplementary Figure 12. Knockdown of IP3R rescues the PD-related phenotypes of PINK1 and Parkin mutants in *Drosophila* (Related to Figure 5).

a, Relative mRNA levels of IP3R in *Drosophila* with indicated genotypes. Three independent experiments were conducted and were quantified. **b-c**, Images of *Drosophila* thoracic (top panels) and wing posture (bottom panels) phenotypes (**b**) and percentages of the flies having abnormal phenotypes (**c**). IP3Ri indicates RNAi knockdown of the *Drosophila* IP3R gene, *itpr*. **d**, Immunofluorescence images of the adult flight muscles (top). Green (mitochondria) and red (actin filament). Scale bars, 5 µm. TUNEL assays of the adult flight muscles (bottom). Green (TUNEL) and blue (DAPI). Scale bars, 5 µm. **e**, Quantification of the percentage of flies having swollen mitochondria. n = 10. **f**, Quantification of the percentage of flies showing apoptotic responses. n = 10. **g**, Measurement of the climbing ability in the adult flies with indicated genotypes. n = 10. **h-i**, Immunofluorescence images (**h**) and numbers (**i**) of DA neurons in PPM1/2 (top), and PPL1 (bottom) regions of adult fly brains. Green (DA neurons).

Images of PPL1 regions were obtained from one of the left or right side of the PPL1 regions. Numbers of the DA neurons in the PPL1 regions were counted from both hemispheres. $n = 10$. Scale bars, 20 µm. One-way ANOVA with Tukey's multiple comparisons test was used (**a**, **e**, **f**, **g**, and **i**). For statistical analysis, one-sided Chi-square test was used (**c**). **** represents $p < 0.0001$, *** represents $p < 0.001$, and ns represents not significant. Source data and the exact *p* values are included within the Source Data file. All data are presented as mean ± SD.

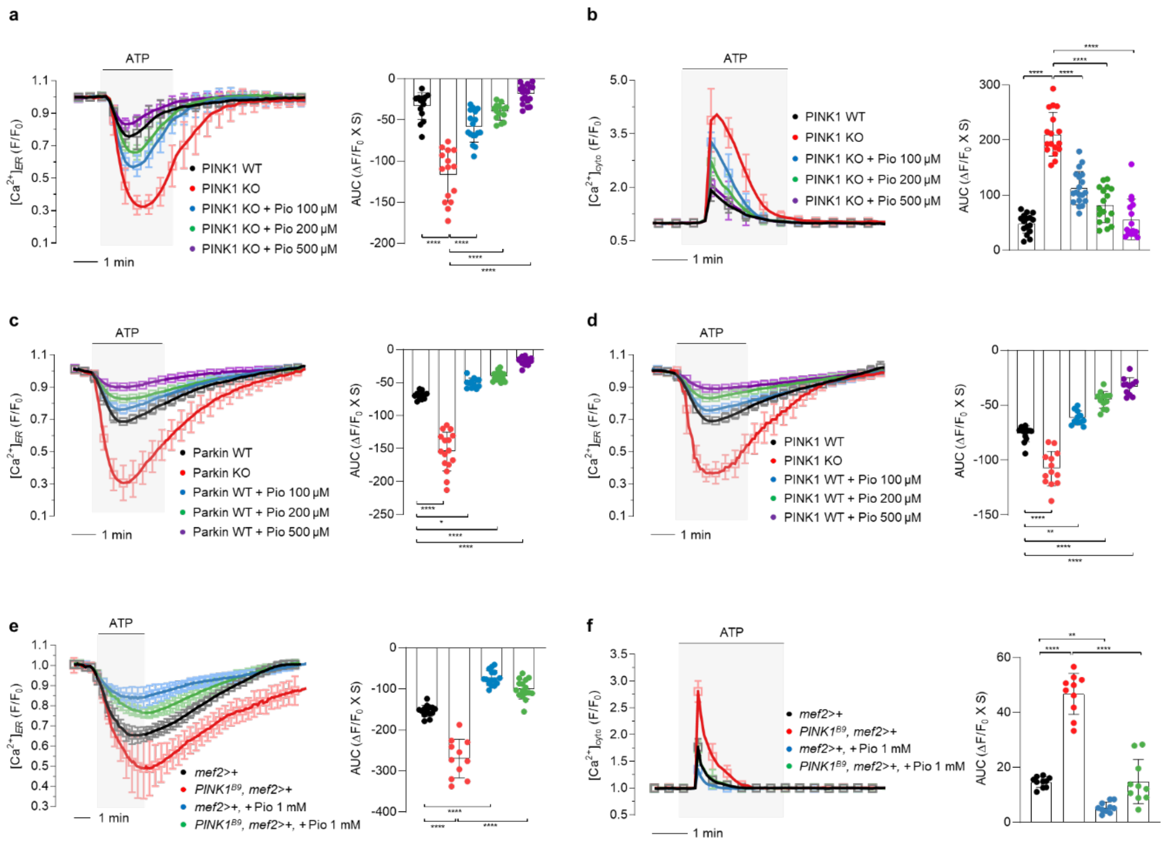

Supplementary Figure 13. Pioglitazone reverses and rescues the increased ER calcium release in PINK1-deficient flies and mammalian cells (Related to Figure 7).

a-b, Measurement of ER (**a**) and cytosolic (**b**) calcium modulations in WT (black) and PINK1 KO (red) MEF cells when treated with indicated concentrations of pioglitazone (blue, green, and purple). The right side bar graphs indicate the quantification of the normalized calcium traces using AUC of calcium release during ATP treatment. *n* = 108~131 cells. **c-d,** Measurement of ER modulations in WT (black) and Parkin KO (red, **c**) or PINK1 KO (red, **d**) MEF cells when treated with indicated concentrations of pioglitazone (blue, green, and purple) in Parkin WT (**c**) or PINK1 WT (**d**) MEF cells. The right side bar graphs indicate the quantification of the normalized calcium traces using AUC of calcium release during ATP treatment. *n* = 111~147 cells. **e-f**, Measurement of ER (**e**) and cytosolic (**f**) calcium modulation in control (*mef2*>+, black) and PINK1 null mutants (*PINK1*^{B9}, red) when treated with 1 mM pioglitazone (blue and green). The right side bar graphs indicate the quantification of the normalized calcium traces using AUC of calcium release during ATP treatment. *n* = 11 ~ 16 flies (**e**). *n* = 10 flies (**f**). Three

independent experiments were conducted and were quantified (**a**-**f**). All statistical significances were determined using one-way analysis of variance (ANOVA) with Tukey's multiple comparisons test. **** represents $p < 0.0001$, ** represents $p < 0.01$, and * represents $p < 0.05$. Source data, the exact p values, and n number of each experiment are included within the Source Data file. All data are presented as mean ± SD.

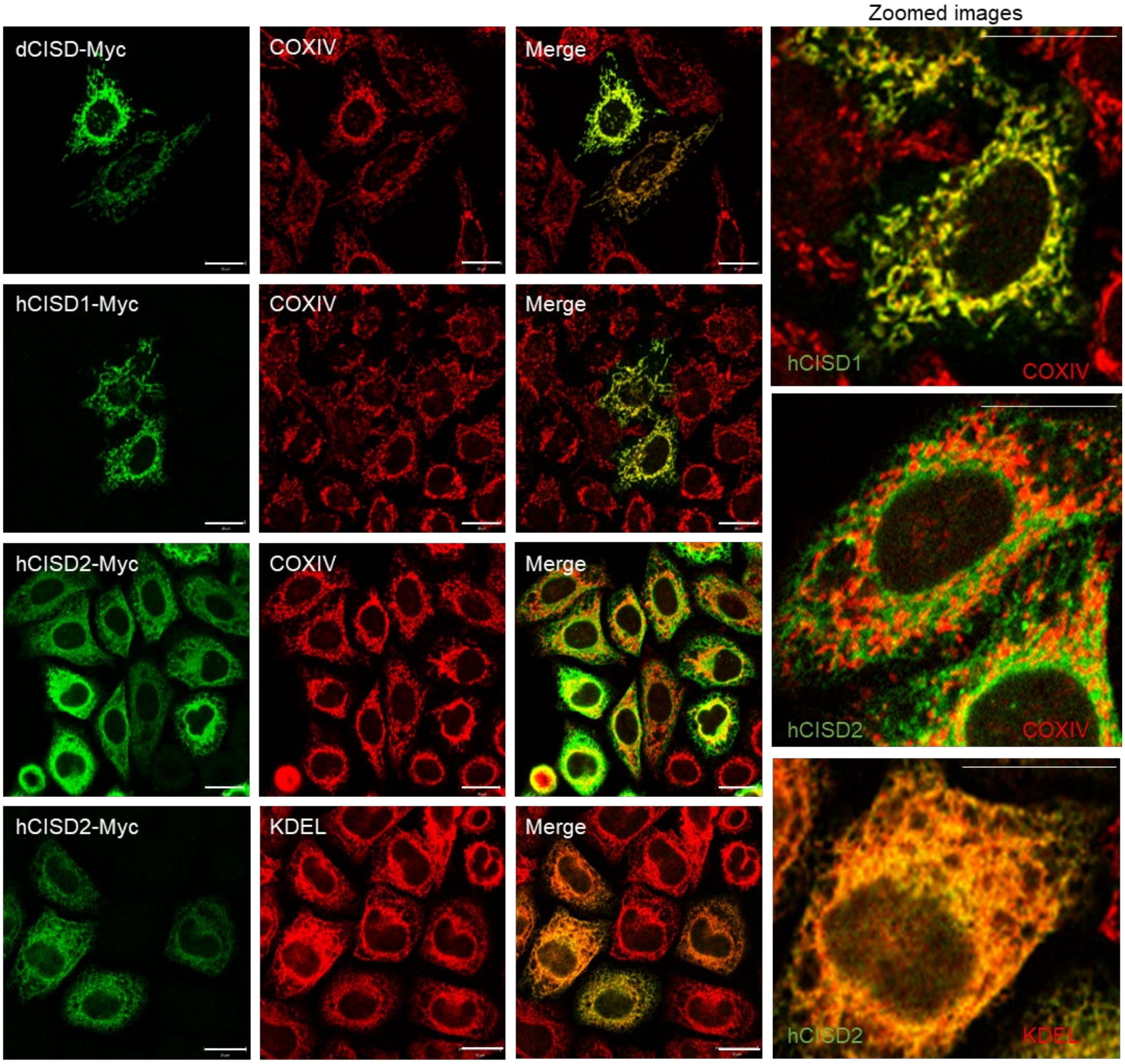

Supplementary Figure 14. Different subcellular localization of human CISD1, human CISD2, and Drosophila CISD (Related to Discussion).

Confocal images of human CISD1 (hCISD1), human CISD2 (hCISD2), and *Drosophila* CISD (dCISD). HeLa cells were transfected with Myc-tagged hCISD1, hCISD2, or dCISD. Zoomed images showed the co-localization of hCISD1 and COXIV (top) and hCISD2 and KDEL (bottom). Green (CISD proteins) and red (COXIV, mitochondrial marker or KDEL, ER marker). Three independent experiments were conducted and were quantified (n = 30 cells). Scale bars indicate 20 μm. The quantification graph and the exact p values are provided as the Source Data file.

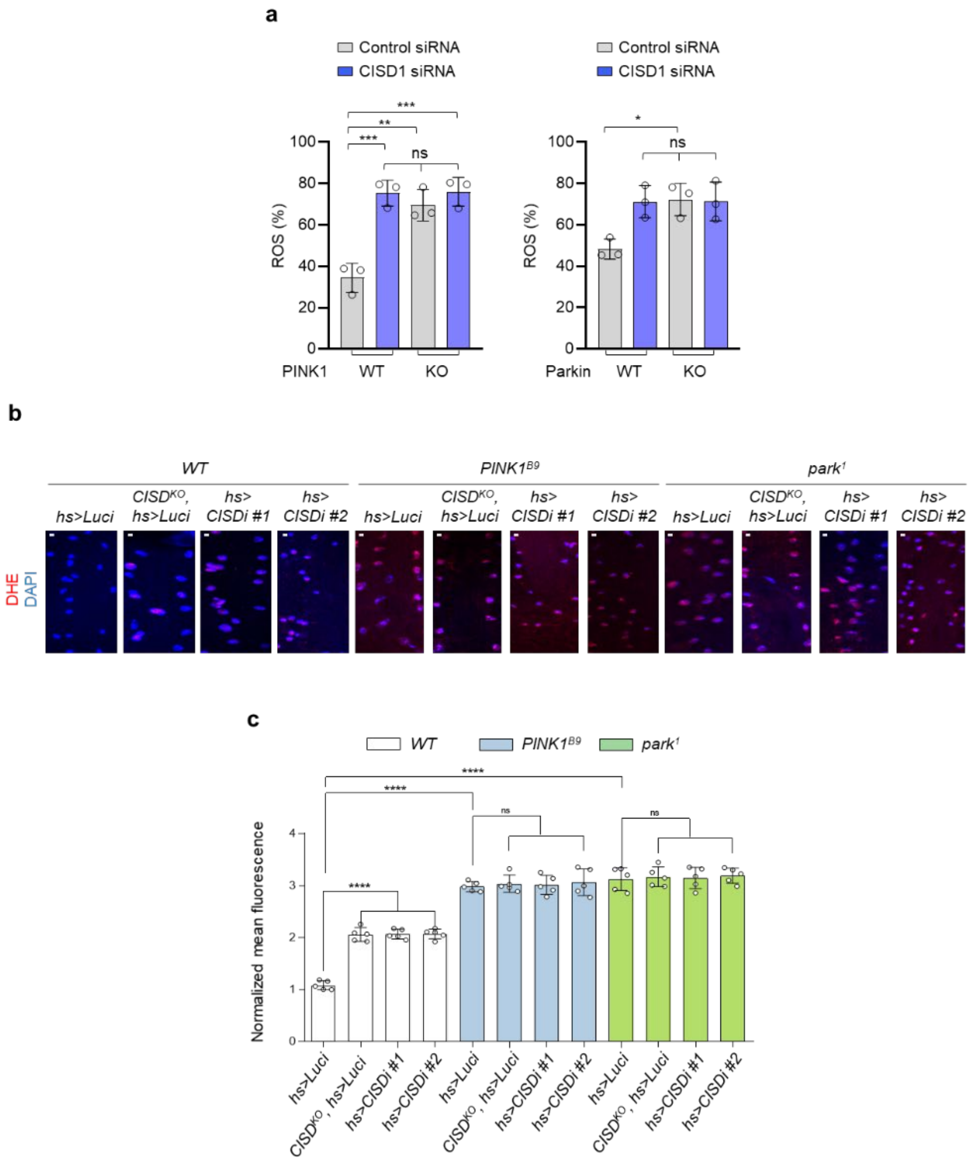

Supplementary Figure 15. ROS levels in mammalian cells and *Drosophila* (Related to Discussion).

a, ROS levels in PINK1 and Parkin WT or KO MEF cells. PINK1 WT and KO MEF cells (left) and Parkin WT and KO MEF cells (right) were transfected with control siRNA (grey) or CISD1 siRNA (blue). Cells were stained with the ROS indicator CM-H_2DCF dye and analyzed using FACS. Bar graphs indicated the percentage of cells having positive CM-H_2DCF signals. Three independent experiments were conducted and were quantified. **b-c**, Images (**b**) and quantification (**c**) of the DHE staining of the adult flight muscles with indicated genotypes. $n = 5$. Red (DHE) and blue (DAPI). Scale bars, 5 µm. One-way ANOVA with Tukey's multiple comparisons test were used (**a** and **c**). ****

represents $p < 0.0001$, *** represents $p < 0.001$, ** represents $p < 0.01$, * represents $p < 0.05$, and ns represents not significant. Source data and the exact p values are included within the Source Data file. All data are presented as mean ± SD.

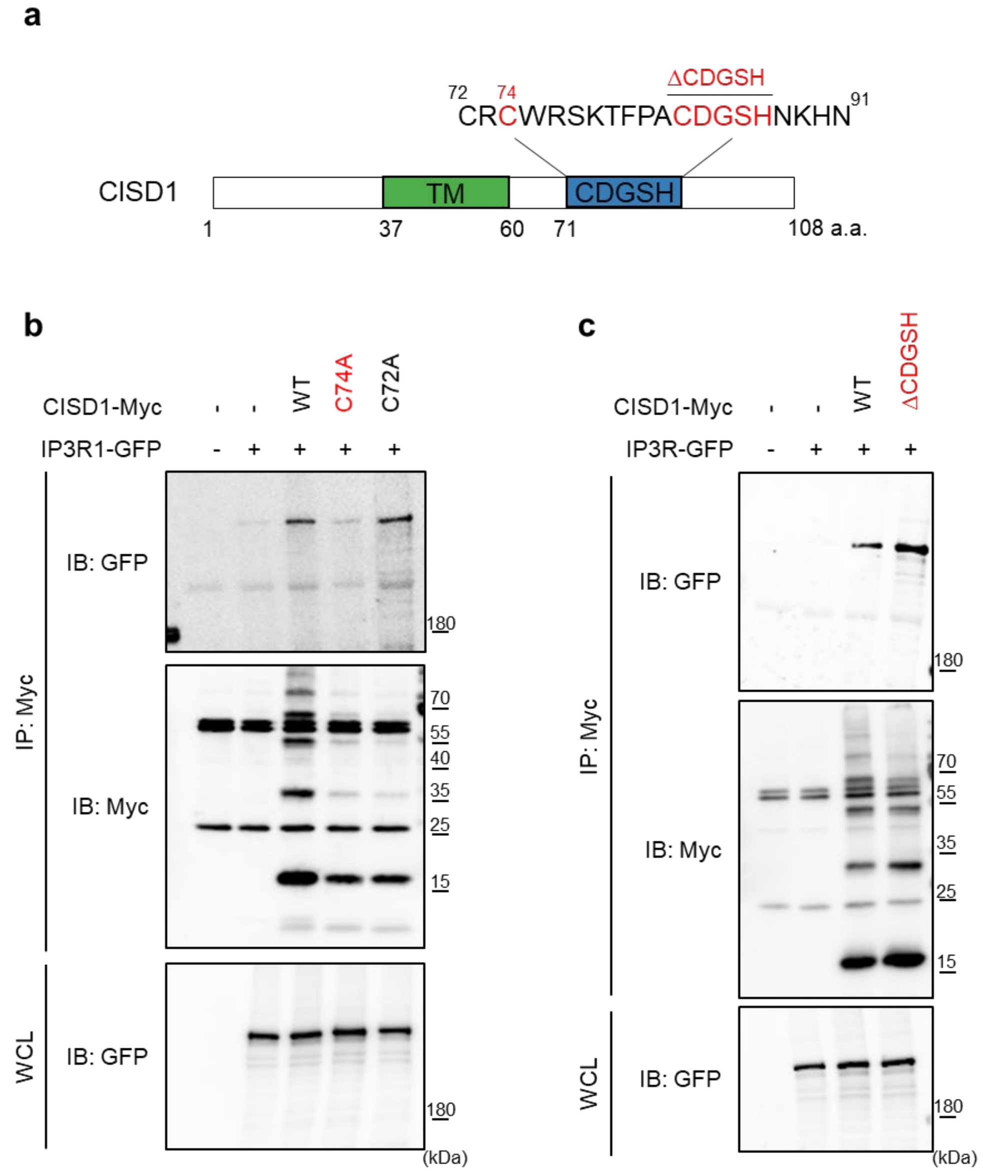

Supplementary Figure 16. Interaction between CISD1 and IP$_3$R1 (Related to Discussion).

a, The domain architecture of human CISD1. The transmembrane domain (TM) and the CDGSH domain are indicated. We generated the CISD1 C72A, C74A, and CDGSH deletion (ΔCDGSH) mutants. **b-c**, HEK293T cells were transfected as indicated and cell lysates were subjected to anti-Myc immunoprecipitation followed by immunoblot analysis. Three independent experiments were conducted and were quantified. Source data and the quantification graphs are provided as the Source Data file.

Supplementary Table 1. List of genes encoding the proteins that are located in the ER or mitochondrial membrane among the substrates of Parkin

Gene	Flybase	Human homolog gene	RNAi#
CISD	CG1458	CISD1, 2	v33925
porin	CG6647	VDAC1, 2, 3	v330138
Cth	CG5345	CTH	v103779
eIF2B-beta	CG2677	EIF2B2	v25426
AlaRS	CG13391	AARS1	v39776
Sply	CG8946	SGPL1	v103485
rdhB	CG7077	DHRS11	v102434
AdSS	CG17273	ADSS, ADSSL1	v29520
Tom70	CG6756	TOMM70	v18112
Pgls	CG17333	PGLS	v100734
Vps4	CG6842	VPS4A, B	v105977
Syx5	CG4214	STX5	v108928
Mpcp2	CG4994	SLC25A3	v101316
Vps35	CG5625	VPS35	v22180
eIF2-alpha	CG9946	EIF2S1	v7798
CG11396	CG11396	TRAPPC12	v103546
sesB	CG16944	SLC25A4, 5, 6, 31	v104576
CG13887	CG13887	BCAP31, 29	v106452
Rdh1	CG2064	RDH11	v103276
CG8839	CG8839	FAAH2	v104479
Vha26	CG1088	ATP6V1E1, 2	v102378
Cyp4p1	CG10842	CYP4V2, A22, B1, F11, F22, CYP20A1	v3331
Idh	CG7176	IDH1, 2	v100554
ALiX	CG12876	PDCD6IP	v32047
Pp2B-14D	CG9842	PPP3CA, B, C	v46873
CG4199	CG4199	AIFM3	v26424
Cat	CG6871	CAT	v103591
Pdh	CG4899	HPGD	v104428
Vha55	CG17369	ATP6V1B2, 1	v46554
CCT4	CG5525	CCT4	v106099
Vha44	CG8048	ATP6V1C1, 2	v101527
Ifrd1	CG31694	IFRD1, 2	v21398

All RNAi lines for candidate genes were obtained from the Vienna Drosophila Stock Center (VDRC) and the lines were denoted as vstock#.

Supplementary Table 2. Antibodies and commercial compounds information

Reagent type	Designation	Source (catalog number, clone numbers, and dillution for antibodies)
Antibody, western blot	GST	Cell Signaling Technology #2625, monoclonal (91G1), 1:1,000
Antibody, western blot	HA	Cell Signaling Technology #3724, monoclonal (C29F4), 1:1,000
Antibody, western blot	GFP	Santa Cruz Biotechnology, SC-9996, monoclonal (B-2), 1:1,000
Antibody, western blot	Tubulin	DSHB, monoclonal (E7), 1:5,000
Antibody, western blot	CaMKI T177	Santa Cruz Biotechnology, SC-28438-R, polyclonal, 1:1,000
Antibody, western blot	CamKII T286	Santa Cruz Biotechnology, SC-12886-R, polyclonal, 1:1,000
Antibody, western blot	CISD1	Proteintech, 16006-1-AP, polyclonal, 1:1,000
Antibody, western blot	Myc	MBL, M192-3, monoclonal (My3), 1:1,000
Antibody, western blot	COXIV	Cell Signaling Technology #4850, monoclonal (3E11), 1:1,000
Antibody, immunoprecipitation	Myc	MBL, M192-3, monoclonal (My3), 1:200
Antibody, immunofluorescence	COXIV	Cell Signaling Technology #4850, monoclonal (3E11), 1:100
Antibody, immunofluorescence	KDEL	Abcam, ab176333, monoclonal (EPR12668), 1:100
Secondary antibody (WB)	HRP-rabbit	Jackson ImmunoResearch #111-035-144, polyclonal, 1:5,000
Secondary antibody (WB)	HRP-mouse	Jackson ImmunoResearch #115-035-146, polyclonal, 1:5,000
Secondary antibody (IF)	TRITC-rabbit	Jackson ImmunoResearch #111-296-144, polyclonal, 1:100
Secondary antibody (IF)	TRITC-mouse	Jackson ImmunoResearch #115-296-146, polyclonal, 1:100
Secondary antibody (IF)	FITC-mouse	Jackson ImmunoResearch #115-096-146, polyclonal, 1:100
Antibody, immunofluorescence	Tyrosine Hydroxylase (TH)	ImmnoStar #22941, monoclonal (LNC1), 1:200
fluorescence dye	Hoechst	Invitrogen #H3569, 1:200
fluorescence dye	Streptavidin	Invitrogen #434301, 1:200
fluorescence dye	phalloidin	Merck #P1951, 1:200
fluorescence dye	DHE	Invitrogen #D1168
commercial compound	TG	Sigma #T9033
commercial compound	2APB	Sigma #D9754
commercial compound	β-escin	Sigma #E1378
commercial compound	IP_3	Sigma #850115P
commercial compound	SlowFade	Invitrogen #S36936
commercial compound	CCCP	Sigma #C2759
commercial compound	MG132	Millipore #474790
commercial compound	CHX	Sigma #0934
