# Supplementary material for: PINK1 and Parkin regulate IP3R-mediated ER calcium release
Source: Nat Commun. 2023 Aug 25;14:5202. doi: 10.1038/s41467-023-40929-z (PMC10457342; doi:10.1038/s41467-023-40929-z)
Supplement: Supplementary file 2 — Reporting Summary [file 41467_2023_40929_MOESM2_ESM.pdf]

nature portfolio

Corresponding author(s): Jongkyeong Chung

Last updated by author(s): Aug 2, 2023

Reporting Summary

Nature Portfolio wishes to improve the reproducibility of the work that we publish. This form provides structure for consistency and transparency in reporting. For further information on Nature Portfolio policies, see our Editorial Policies and the Editorial Policy Checklist.

Statistics

For all statistical analyses, confirm that the following items are present in the figure legend, table legend, main text, or Methods section.

n/a	Confirmed	
☐	☒	The exact sample size (n) for each experimental group/condition, given as a discrete number and unit of measurement
☐	☒	A statement on whether measurements were taken from distinct samples or whether the same sample was measured repeatedly
☐	☒	The statistical test(s) used AND whether they are one- or two-sided<br>*Only common tests should be described solely by name; describe more complex techniques in the Methods section.*
☐	☒	A description of all covariates tested
☐	☒	A description of any assumptions or corrections, such as tests of normality and adjustment for multiple comparisons
☐	☒	A full description of the statistical parameters including central tendency (e.g. means) or other basic estimates (e.g. regression coefficient) AND variation (e.g. standard deviation) or associated estimates of uncertainty (e.g. confidence intervals)
☐	☒	For null hypothesis testing, the test statistic (e.g. F, t, r) with confidence intervals, effect sizes, degrees of freedom and P value noted<br>*Give P values as exact values whenever suitable.*
☒	☐	For Bayesian analysis, information on the choice of priors and Markov chain Monte Carlo settings
☒	☐	For hierarchical and complex designs, identification of the appropriate level for tests and full reporting of outcomes
☒	☐	Estimates of effect sizes (e.g. Cohen's d, Pearson's r), indicating how they were calculated

Our web collection on statistics for biologists contains articles on many of the points above.

Software and code

Policy information about availability of computer code

Data collection	CISD1 3D structures was displayed using PyMOL v2.4.0.<br>Basal cytosolic calcium levels and ROS levels were collected using FACS canto II instrument (BD Biosciences).<br>Calcium measurement raw data in cells were collected using confocal microscopy (Carl Zeiss, LSM710).<br>Calcium measurement raw data in Drosophila were collected using IX-73 inverted microscope (Olympus, Japan) with a camera (Prime-BSI CMOS camera, Teledyne Photometrics) attachment and an illuminator (pe-340Fura, CoolLED, UK).<br>Immunohistochemistry raw data in Drosophila were collected using confocal microscopy (Carl Zeiss, LSM710).
Data analysis	In general, results presented as mean SD were collected and calculated using GraphPad Prism (v8.0).<br>For FACS analysis, raw datasets were analyzed by BD FACSDiva v.6.1.3 software.<br>For immunoblotting, raw datasets were analyzed using Image J v1.51 and Multi gauge v3.0.<br>For immunohisochemistry in Drosophila, raw datasets were analyzed using Zen imaging software v7.0.<br>For calcium measurement in cell, raw datasets were analyzed using Zen imaging software v7.0.<br>For calcium measurement in Drosophila, raw datasets were analyzed using Metafluor software (Molecular Devices, v6.3).

For manuscripts utilizing custom algorithms or software that are central to the research but not yet described in published literature, software must be made available to editors and reviewers. We strongly encourage code deposition in a community repository (e.g. GitHub). See the Nature Portfolio guidelines for submitting code & software for further information.

Data

Policy information about availability of data

All manuscripts must include a data availability statement. This statement should provide the following information, where applicable:

- Accession codes, unique identifiers, or web links for publicly available datasets
- A description of any restrictions on data availability
- For clinical datasets or third party data, please ensure that the statement adheres to our policy

All data needed to evaluate the conclusions of paper are presented within the paper and the supplementary materials.

Human research participants

Policy information about studies involving human research participants and Sex and Gender in Research.

Reporting on sex and gender	N/A
Population characteristics	N/A
Recruitment	N/A
Ethics oversight	N/A

Note that full information on the approval of the study protocol must also be provided in the manuscript.

Field-specific reporting

Please select the one below that is the best fit for your research. If you are not sure, read the appropriate sections before making your selection.

☒ Life sciences ☐ Behavioural & social sciences ☐ Ecological, evolutionary & environmental sciences

For a reference copy of the document with all sections, see nature.com/documents/nr-reporting-summary-flat.pdf

Life sciences study design

All studies must disclose on these points even when the disclosure is negative.

Sample size

Sample size was determined based on standards for experimental cell biology and Drosophila studies, attempting to have a minimum of n = 3 biological replicates with sufficient reproducibility. All experiments were performed more than three independent repeats in every case to ensure reproducibility. In the cell calcium measurement experiment, we also conducted three dependent experiments and analyzed more than 50 cells to ensure reproducibility under healthy cell conditions.

Exceptions where n=2: Supplementary Fig. 3g-l, for endogenous CISD1 protein levels in Drosophila samples, the observation were validated across two independent, providing enough confidence on the conclusions;
In Supplementary Fig.2b-c, we measured n= 7 flies for Drosophila ER calcium measurement, and these experiments obtained the same results with 7 flies, demonstrating sufficient reproducibility. Regarding the calcium measurement results of the CISD RNAi (v33925) line, which showed positive results in the calcium screen in Drosophila, we conducted three independent experiments using two lines of CISD RNAi (v104501 and v33925) (Figure 2b, Supplementary Fig. 4, Supplementary Fig. 5). Although the screen results were obtained from a single experiment, we replicated the calcium measurements in the two lines at least three times in different experiments, demonstrating sufficient reproducibility to support the conclusions of our study.

The sample size selection for both in vitro and in vivo experiments was based on the lab's previous experience.
The following references were used as a basis for determining the sample size:
1. Cell biology experiments: Loss of UCHL1 rescues the defects related to Parkinson's disease by suppressing glycolysis. Sci Adv 7 (2021).
2. Drosophila experiments: Mitochondrial dysfunction in Drosophila PINK1 mutants is complemented by parkin. Nature 441, 1157-1161 (2006).
3. Calcium measurement experiments in Drosophila: Mitochondrial calcium uniporter in Drosophila transfers calcium between the endoplasmic reticulum and mitochondria in oxidative stress-induced cell death. J Biol Chem 292, 14473-14485 (2017)

The sample size selection for calcium imaging experiments in cells was based on previous studies,
1. Measurement of calcium levels in mammalian cells: ANKTM1, a TRP-like channel expressed in nociceptive neurons, is activated by cold temperatures. Cell, 112: 819-829 (2003).
2. Measurement of the influx and efflux of ER calcium in mammalian cells: ER-mitochondria cross-talk is regulated by the Ca2+ sensor NCS1 and is impaired in Wolfram syndrome. SCIENCE SIGNALING, Vol 11, Issue 553 (2018).

Data exclusions

There were no data exclusions, except failed immunoblots that could not be quantified.

Replication

Each data involved at least three independent experiments. For quantitative measurements, three or more independent experiments were carried out and statistical analyses were followed. All attempts for replication were successful.

Randomization

Samples were allocated randomly for culture and analysis in the mammalian cell system. Flies were randomly chosen for each experiment. For both in vitro and in vivo, samples were allocated based on the corresponding genotypes. Randomization was done by randomly capturing imaging regions in fluorescence microscopic analyses and calcium measurement of cells and Drosophila. In other experiments, unbiased quantifications were performed using data analyzers (such as Image J and Multi gauge).

Blinding

Blinding was not required for this study because there were no data exclusions and conclusions on objective quantitative analysis of data.

Reporting for specific materials, systems and methods

We require information from authors about some types of materials, experimental systems and methods used in many studies. Here, indicate whether each material, system or method listed is relevant to your study. If you are not sure if a list item applies to your research, read the appropriate section before selecting a response.

Materials & experimental systems

n/a	Involved in the study
☐	☒ Antibodies
☐	☒ Eukaryotic cell lines
☒	☐ Palaeontology and archaeology
☐	☒ Animals and other organisms
☒	☐ Clinical data
☒	☐ Dual use research of concern

Methods

n/a	Involved in the study
☒	☐ ChIP-seq
☒	☐ Flow cytometry
☒	☐ MRI-based neuroimaging

Antibodies

Antibodies used

We used antibodies in the following list:
Antibody, western blot GST, Cell Signaling Technology #2625, monoclonal (91G1), 1:1,000
Antibody, western blot HA, Cell Signaling Technology #3724, monoclonal (C29F4), 1:1,000
Antibody, western blot GFP, Santa Cruz Biotechnology, SC-9996, monoclonal (B-2), 1:1,000
Antibody, western blot Tubulin, DSHB, monoclonal (E7), 1:5,000
Antibody, western blot CaMKI pT177, Santa Cruz Biotechnology, SC-28438-R, polyclonal, 1:1,000
Antibody, western blot CamKII pT286, Santa Cruz Biotechnology, SC-12886-R, polyclonal, 1:1,000
Antibody, western blot CISD1, Proteintech, 16006-1-AP, polyclonal, 1:1,000
Antibody, western blot Myc, MBL, M192-3, monoclonal (My3), 1:1,000
Antibody, western blot COXIV, Cell Signaling Technology #4850, monoclonal (3E11), 1:1,000
Antibody, immunoprecipitation Myc, MBL, M192-3, monoclonal (My3), 1:200
Antibody, immunofluorescence COXIV, Cell Signaling Technology #4850, monoclonal (3E11), 1:100
Antibody, immunofluorescence KDEL, Abcam, ab176333, monoclonal (EPR12668), 1:100
Secondary antibody (WB) HRP-rabbit, Jackson ImmunoResearch #111-035-144, polyclonal, 1:5,000
Secondary antibody (WB) HRP-mouse, Jackson ImmunoResearch #115-035-146, polyclonal, 1:5,000
Secondary antibody (IF) TRITC-rabbit, Jackson ImmunoResearch #111-296-144, polyclonal, 1:100
Secondary antibody (IF) TRITC-mouse, Jackson ImmunoResearch #115-296-146, polyclonal, 1:100
Secondary antibody (IF) FITC-mouse, Jackson ImmunoResearch #115-096-146, polyclonal, 1:100
Antibody, immunofluorescence tyrosine hydroxylase (TH), ImmnoStar #22941, monoclonal (LNC1), 1:200

Validation

All antibodies used in this study have been validated by the manufacturers or the original authors of previous publications.

Antibody, western blot GST, Cell Signaling Technology #2625: 140 citations reported on manufacturer's website (https://www.cellsignal.com/products/primary-antibodies/gst-91g1-rabbit-mab/2625)
Antibody, western blot HA, Cell Signaling Technology #3724: 2406 citations reported on manufacturer's website (https://www.cellsignal.com/products/primary-antibodies/ha-tag-c29f4-rabbit-mab/3724)
Antibody, western blot GFP, Santa Cruz Biotechnology, SC-9996: 11 references reported on manufacturer's website (https://www.scbt.com/p/gfp-antibody-b-2?gclid=CjwKCAjwt52mBhB5EiwA05YKo7kvHZjt2D1eY8k1jjcluIpeFjlzOLncr-xaYoxtniB15GRtHDgOMxoCC9wQAvD_BwE)
Antibody, western blot Tubulin, DSHB: 130 references reported on manufacturer's website (https://dshb.biology.uiowa.edu/E7_2)
Antibody, western blot CaMKI pT177, Santa Cruz Biotechnology, SC-28438-R: excellent specificity on manufacturer's website (https://www.lsbio.com/antibodies/camk1-antibody-camki-antibody-elisa-if-immunofluorescence-wb-western-ls-c500740/514254?adid=10134&gclid=CjwKCAjwt52mBhB5EiwA05YKo0TY0HYC0ck90a1QftasOpdK16Solm6935VS0xi7T1saEaKMHHs_aRoCOYoQAvD_BwE#validation-section)
Antibody, western blot CamKII pT286, Santa Cruz Biotechnology, SC-12886-R: excellent specificity and optimization for IHC reported on manufacturer's website (https://www.lsbio.com/antibodies/camk2-antibody-camkii-antibody-phospho-thr286-ihc-wb-western-ihc-plus-ls-b2637/87043?adid=10134&gclid=CjwKCAjwt52mBhB5EiwA05YKo23uf5Kx0foJaxYM0Uy0YRPlWBdYG5Xv9QN24Z_We0HK7Q7EsXTmqxoC75oQAvD_BwE#validation-section)
Antibody, western blot CISD1, Proteintech, 16006-1-AP: 35 publications reported on manufacturer's website (https://

www.ptglab.com/products/mitoNEET,CISD1-Antibody-16006-1-AP.htm)
Antibody, western blot Myc, MBL, M192-3: 36 citations reported on manufacturer's website (https://www.mblbio.com/bio/g/dtl/A/?pcd=M192-3)
Antibody, western blot COXIV, Cell Signaling Technology #4850: 512 citations reported on manufacturer's website (https://www.cellsignal.com/products/primary-antibodies/cox-iv-3e11-rabbit-mab/4850)
Antibody, immunofluorescence KDEL, Abcam, ab176333: 16 references reported on manufacturer's website (https://www.abcam.com/products/primary-antibodies/kdel-antibody-epr12668-ab176333.html)
Antibody, immunofluorescence tyrosine hydroxylase (TH), ImmnoStar #22941: 1802 citations reported on manufacturer's website (https://www.immunostar.com/product/tyrosine-hydroxylase-antibody/)

Eukaryotic cell lines

Policy information about cell lines and Sex and Gender in Research

Cell line source(s)	Commercial cell lines: HeLa (ATCC #CCL-2)<br>HEK293 and HEK293T cells were from Dr John Blenis at Cornell Medical School.<br>PINK1 WT and KO mouse embryonic fibroblast , and Parkin WT and KO mouse embryonic fibroblast were from Dr Young-Yun Kong at Seoul National University.<br>CISD1 WT mouse embryonic fibroblast from Dr Jae Ung Jung at University of Southern California (currently Cleveland Clinic).<br>CISD1 KO mouse embryonic fibroblast was generated in this study using CRISPR-Cas9 system from the above WT mouse embryonic fibroblast obtained from Dr Jung.
Authentication	Cell lines were authenticated using morphology and immunoblot analyses, and were carefully labeled and tracked in this lab.
Mycoplasma contamination	Cells used for all experiments were mycoplasma negative.
Commonly misidentified lines<br>(See ICLAC register)	None of commonly misidentified cell lines has been used.

Animals and other research organisms

Policy information about studies involving animals; ARRIVE guidelines recommended for reporting animal research, and Sex and Gender in Research

Laboratory animals	The laboratory animal involved in the study is Drosophila melanogaster. Only male animals were used for all experiments. Drosophila lines used in the experiments were hs-GAL4 (2077; the Bloomington Drosophila Stock Center), mef2-GAL4 (27390; the Bloomington Drosophila Stock Center), PINK1B9 (34749; the Bloomington Drosophila Stock Center), park1 (34747; the Bloomington Drosophila Stock Center), UAS-CISD RNAi (33925 and 104501; the Vienna Drosophila Resource Center), UAS-Itpr (30742; the Bloomington Drosophila Stock Center), UAS-Itpr RNAi (6484; the Vienna Drosophila Resource Center), UAS-ERGCaMP6-210 (83294; the Bloomington Drosophila Stock Center), UAS-GCaMP5G (42037; the Bloomington Drosophila Stock Center), UAS-Luciferase RNAi (31603; the Bloomington Drosophila Stock Center), and UAS-DsRed (6282; the Bloomington Drosophila Stock Center). The UAS-CISD WT-HA fly line was generated by microinjecting pUAST-CISD-HA into w1118 embryos in this study.<br><br>Wandering third instar larvae were used for Drosophila calcium imaging. For all experiments investigating PD-related phenotypes, including thorax, wing, climbing, IHC, TUNEL, and ROS, 3-day-old male flies were used. Meanwhile, for observing the DA neuron, 30-day-old male flies were employed. This experimental approach was applied uniformly to all genotypes.<br><br>We used RNAi lines from the Vienna Drosophila Resource Center for measurement of ER calcium release in Drosophila.<br>The RNAi lines include v330138, v103779, v25426, v18112, v100734, v22180, v7798, v104576, v103276, v104479, v3331, v100554, v32047, v26424, v46554, v101527, and v21398.
Wild animals	No wild animals were used in the study.
Reporting on sex	Males were used in all Drosophila experiments.
Field-collected samples	No field-collected samples were used in the study.
Ethics oversight	No ethical approval is needed for experiments using Drosophila.

Note that full information on the approval of the study protocol must also be provided in the manuscript.
